# Synthesis and characterization of bi-functional Co-Ag MOF@CuO nanorods as an innovative robust heterogeneous catalytic material for the fabrication of fused 1,4-dihydropyridine derivatives

**DOI:** 10.1038/s41598-026-43843-8

**Published:** 2026-05-18

**Authors:** Negar Hoot, Enayatollah Sheikhhosseini, Sayed Ali Ahmadi, Mahdieh Yahyazadehfar

**Affiliations:** Department of Chemistry, Islamic Azad University, Ke.C, Kerman, Iran

**Keywords:** Chemistry, Materials science

## Abstract

This study used the co-precipitation method to produce novel nanorods from bi-functional Co-Ag metal-organic frameworks (BF Co-Ag MOF). CuO was then immobilized onto the BF Co-Ag MOF to create a new heterogeneous catalytic material (BF Co-Ag MOF@CuO). This material was thoroughly examined using several analytical methods, including field-emission scanning electron microscopy (FE-SEM), energy-dispersive X-ray spectroscopy (EDX), transmission electron microscopy (TEM), X-ray diffraction (XRD), N_2_ adsorption-desorption, vibrating-sample magnetometry (VSM), and Fourier transform infrared spectroscopy (FT-IR). The one-pot, multi-component Hantzsch fabrication of biologically significant fused 1,4-dihydropyridines (1,4-DHPs) was used to assess the synthesized nanorods’ catalytic efficiency. Dimedone, commercially available aldehydes, and ammonium acetate as a nitrogen source were used in this chemical reaction at 60 °C in aqueous media. ^1^H-NMR and^13^ C-NMR spectroscopy were used to thoroughly analyze the obtained products. The quick fabrication of a wide variety of 1,4-DHPs with short chemical transformation times, straightforward experimental and work-up processes, simple catalytic material preparation, outstanding catalytic performance, notable product outputs, an eco-friendly solvent, and reusability are just a few benefits of the suggested approach.

**Supplementary Information:**

The online version contains supplementary material available at 10.1038/s41598-026-43843-8.

## Introduction

Because of their coordinative sites and high surface-to-volume ratio, which provide more active sites per unit area than traditional heterogeneous catalytic materials, metal oxide nanoparticles are widely used as highly effective catalysts in a variety of organic chemical transformations, because of their affordability, accessibility, high catalytic efficiency, and rapid chemical transformation rates, CuO nanoparticles (CuO NPs) have attracted greater attention than conventional catalytic materials^1^. Nevertheless, promoters, fabrication process optimization, or the creation of CuO-based composites can all help reduce the sintering and aggregation that copper-based catalytic materials are susceptible to^2,3^.

Metal-organic frameworks (MOFs) have attracted significant interest among materials used to create composites because of their stability, high surface area, and precise control over the shape and size distribution of pores^4^. MOFs are made up of organic ligands that serve as binders and metal ions. They are ideal precursors for the synthesis of homogeneous nanocomposites due to their distinct 3D network structure and adjustable design^5^.

Monometallic MOF catalysts usually exhibit either low activity or poor selectivity. To enhance the catalytic properties of MOFs, it has been proposed to incorporate second metal ions into the framework nodes to prepare bimetallic MOFs^6^. Partial substitution of second-metal ions into the inorganic nodes or secondary-building units (SBUs) of the framework will allow the bimetallic system to exhibit synergistic effects^7^. The synergistic effect between two metal ions to activate the reactants and reduce the reaction energy barrier in heterogeneous catalysis^8,9^. Bimetallic MOFs have shown promising catalytic performance in a variety of reactions, including oxidation and reduction reactions, due to the distortion of the framework that facilitates access to the active sites, thus enhancing the catalyst’s catalytic activity^10^.

In industrial chemistry, the concept of “green chemistry” is increasingly adopted to address important scientific issues related to human health and environmental preservation. In contemporary synthetic organic chemistry, multicomponent chemical transformations have become a very useful and successful strategy. These chemical transformations, which entail several synthetic steps and don’t isolate intermediates, decrease time, energy, and raw material consumption. Chemists are therefore focusing on Multicomponent reactions (MCRs) to synthesize organic compounds. MCRs are an effective tool for creating heteroatom-carbon bonds and generating energy because they greatly improve the selectivity and reactivity of chemical reactions. Among these, the Hantzsch chemical transformation is one of the most well-known MCRs, yielding 1,4-dihydropyridines as its final products^11,12^.

Significant efforts have been made to develop catalytic processes that use water as an environmentally friendly medium for chemical transformations to promote greener fabrication. Certain interactions that improve these processes are facilitated by the special physicochemical characteristics of water, such as hydrogen bonding and the hydrophobic effect. Using heterogeneous catalytic material systems is the best approach to avoid both financial and environmental problems in catalytic material separation. The high surface-to-volume ratio of nanoparticles has attracted significant attention because it allows lower concentrations of catalytic material while maintaining extremely efficient conversion rates, ultimately lowering the costs associated with chemical transformation^13^.

Because of their favorable properties, such as immiscibility with common organic solvents, air stability, non-hygroscopic nature, and thermal and chemical stability, heterogeneous acid-catalytic materials have attracted significant interest in organic transformations. These catalytic materials have been supported on various materials such as metal oxides^14^, periodic mesoporous organosilica^15^, polypropylene fiber^16^, activated carbon^17^, polyethylene glycol^18^, zeolites^19^, and magnetic nanoparticles (MNPs)^20^, among others.

In the pharmaceutical industry, Hantzsch multisubstituted 1,4-dihydropyridines (1,4-DHPs) are an important class of bioactive compounds known for their ability to block Ca^2+^ channels and act as antidiabetic agents to treat cardiovascular diseases, such as hypertension^21^. Numerous biological activities, such as geroprotective, vasodilatory, analgesic, hepatoprotective, sedative, antitumor, antiatherosclerotic, anticonvulsant properties^21,22^, hypnotic, anti-inflammatory, anti-anxiety, antidepressant, antimutagenic, and antiplatelet aggregation qualities are displayed by the heterocyclic ring in DHPs^22–25^.

Quinolones are an important class of calcium channel blockers with a 1,4-dihydropyridine nucleus^25^. These compounds are widely used as vasodilators and calcium channel blockers for the treatment of cardiovascular diseases such as hypertension^26^ and migraines^27^. Additionally, they serve as chemosensitizers in tumor therapy^28^, and cerebral anti-ischemic agents for Alzheimer’s disease^29^. Their pharmacological properties extend to anticonvulsant, analgesic, hepatoprotective, bronchodilatory, antiatherosclerotic, antidepressant^25^, antianxiety^25^, antimalarial^30^, neuroprotective^25^, heptatoprotective^30^, antitumor^25^, antimutagenic, antineoplastic^31^, sedative, anti-inflammatory, hypnotic^25^, and antiplatelet aggregation activities^22–24^, neuroprotectant^32^, platelet antiaggregatory activity^28^, and these moieties also exhibit DNA intercalating^31^. Additionally, certain 1,4-DHP derivatives are being investigated as selective antagonists of G-protein-coupled receptors, adenosine receptor modulators, and potassium and sodium ion channels, with potential uses across a variety of therapeutic domains^33^.

**Fig. 1:**
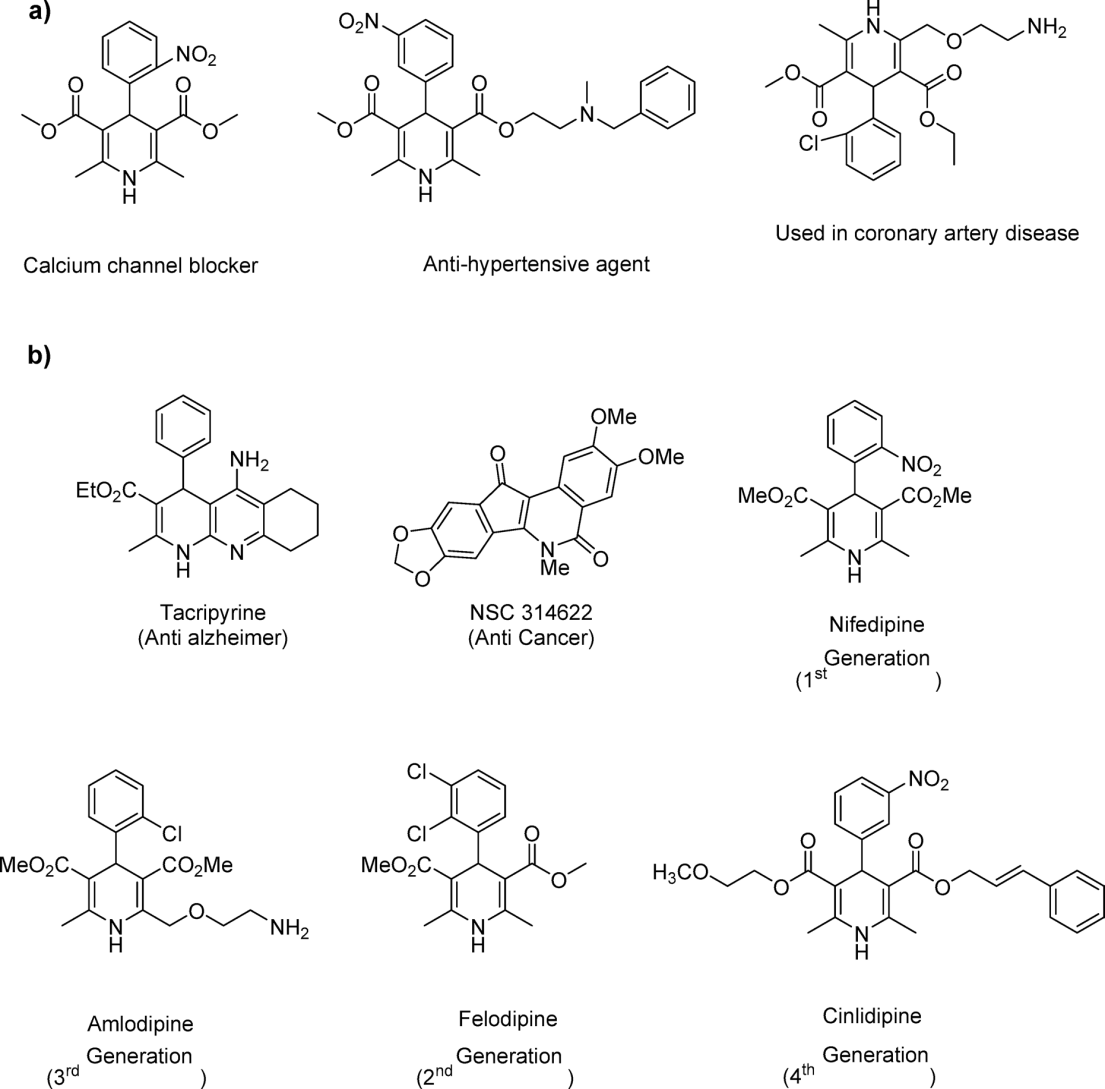
(**a**) Some commercial drugs containing 1,4-dihydropiridine, and (**b**) some biologically active 1,4-DHP molecules.

Due to the remarkable biological activity of the 1,4-dihydropyridine (DHP) core, these compounds serve as the fundamental structure for numerous commercial drugs, including amlodipine, nifedipine, nimodipine, felodipine, isradipine, and nicardipine. The structures of several biologically active 1,4-DHPs are depicted in Fig. 1a^11^, while Fig. 1b illustrates the structures of commercially available DHP-based drugs^34^. In recent years, there has been growing interest in the fabrication of 1,4-dihydropyridine derivatives, leading to the development of various methods for their preparation.

Because 1,4-DHP derivatives have significant pharmacological and biological significance, a variety of techniques have been used to fabricate these compounds under modified conditions and through one-pot multi-component chemical transformation using various techniques/mediums, such as microwave^35^, ionic liquid^36^, high temperature in refluxing solvent^37^, TMSCl-NaI^38^, metal triflates^39^, and CAN^40^ and different catalytic materials such as *p*-dodecylbenzenesulfonic acid (DBSA)^41^, amberlyst-15^42^, [Hmim]TFA^43^, Lproline^44^, MCM-41-SO_3_H^45^, Bronsted acidic imidazolium salts containing perfluoroalkyl tails^46^, perchloric acid adsorbed on magnetic Fe_3_O_4_ nanoparticles coated with silica^47^, magnetic Fe-C-O-Mo alloy nano-rods^48^ etc. However, to the best of our knowledge, these methods have several drawbacks, including low yields, the occurrence of multiple side products, lengthy chemical transformation times, harsh conditions for chemical transformation, and laborious workups, the use of high temperatures, the use of costly catalytic materials, difficulties handling the catalytic material, difficulties separating and reusing the catalytic material, and the use of large amounts of volatile organic solvents. Therefore, it is still necessary to develop a new, flexible, highly effective, easy, affordable, and environmentally friendly method for the preparation of dihydropyridines. This is an active research area, and there is room for improvement using robust heterogeneous catalytic material, leading to milder chemical transformation conditions and higher product yields. In keeping with our earlier work^49–54^, we report an “on-water” fabrication of a wide variety of 1,4-DHPs by a one-pot multi-component chemical transformation of various aromatic aldehydes, ammonium acetate, and dimedone in the presence of the novel, reusable, bi-functional Co-Ag MOF@CuO as a heterogeneous catalytic material in aqueous media at 60 °C. This is because combining a water-based medium with a nanocatalytic material is an appealing way to expand sustainable, green protocols. The mixing of metals in MOF structure is widely recognized as an effective way to achieve optimal catalytic properties. Bi-functional Co–Ag MOF has two different metal ions in its inorganic nodes, arising from the synergistic combination of Lewis-acidic Co^2+^/Ag^+^ metal nodes. Ag^+^ ions contribute redox-active functionality, enhancing electron transfer in the Hantzsch reaction. In addition, the incorporation of second metal ions (Co^2+^) into the MOF structure enhances the stability of the MOF. Therefore, bifunctional Co–Ag MOF exhibits exposed active sites and good stability, enabling it to extend its applications in the catalysis of more challenging reactions. Additionally, immobilized CuO nanoparticles introduce additional active centers. The rod-like morphology and homogeneous particle distribution facilitate efficient catalysis, resulting in excellent yields, short reaction times, and remarkable reusability under mild aqueous conditions.

## Experimental section

### Chemicals and reagent

Silver nitrate, cobalt nitrate, trimesic acid, ammonium acetate, aromatic aldehydes, and dimedone were all supplied by the Merck company and used without further purification.

### Material characterization

A PC-APD X-ray diffractometer with Kα radiation (α_2_, λ_2_ = 1.54439 Å) and graphite monochromatic Cu radiation (α_1_, λ_1_ = 1.54056 Å) (Philips, Netherlands) was used to analyze the phase structure, crystallite size, and crystallinity of BF Co-Ag MOF@CuO nanorods. Software called X’Pert HighScore Plus was used to analyze the data. With a step size of 0.016°, the XRD pattern was recorded over 2–80 ° 2θ. Energy dispersive spectrometry (EDS) (KYKY and EM 3200) and scanning electron microscopy (SEM) were used to analyze the composition and morphology of BF Co-Ag MOF@CuO nanorods. Using a Micromeritics ASAP 2010 analyzer, the Brunauer-Emmett-Teller (BET) and Barrett-Joyner-Halenda (BJH) techniques were used to calculate the nanorods’ surface area and pore-size distribution. A Lakeshore 7407 magnetometer was used to measure magnetization at room temperature while applied magnetic fields were present. Uncorrected melting points were determined using an Electrothermal 9100 device. The solvent was used as a reference, and chemical shifts were reported in parts per million (ppm, δ scale) relative to an internal tetramethylsilane (TMS) standard (0.00 ppm). Using KBr pellets and a Buck 400 scientific spectrometer, infrared (IR) spectra were captured.

### Fabrication of nanocatalytic material

#### Preparation of CuO nanoparticles

First, deionized water was used to prepare a 0.1 M solution of copper (II) sulfate pentahydrate (CuSO_4_·5H_2_O). A saturated NaOH solution was added gradually until the solution reached pH 8.0, at which point precipitates formed. To remove any remaining raw materials, these precipitates were collected and subjected to three ethanol washes. After washing, the items were dried for 12 h at 80 °C. The precipitates were then calcined for an hour at 400°C^55^.

### Synthesis of bi-functional Co-Ag metal organic frameworks (BF Co-Ag MOF)

cobalt (II) nitrate hexahydrate (Co (NO_3_)_2_.6H_2_O) (2.061 mmol, 0.6 g) and silver nitrate hexahydrate (AgNO_3_.6H_2_O) (3.532 mmol, 0.6 g) were dissolved in deionized water separately, added dropwise to a solution containing (3.526 g) of trimesic acid ((TMA) known as 1,3,5-benzenetricarboxylic acid) linker dissolved in deionized water, and the resulting solution was allowed to stir for two hours at 80 °C to complete the BF Co-Ag MOF precipitation process. The final product was dried for 1 h at 130 °C after being cleaned three times with ethanol to remove any residual raw materials.

### Synthesis of BF Co-Ag MOF@CuO nanorods

In a beaker set at 80 °C, a 0.6 g (0.894 mmol) sample of the dried BF Co-Ag MOF was dissolved in deionized water. CuO nanoparticles weighing 0.024 g (0.298 mmol) were then added, and the mixture was stirred for 10 min to produce a homogenous solution. For microwave irradiation, the finished powder mixture was moved to a glass vial. The vial was placed immediately in a 140 W microwave oven and left there for 90 min. The finished products have been cleaned with acetic acid to remove all remaining impurities. The powder was then calcined at 175 °C for 45 min in a furnace.

### General process of preparing fused 1,4-dihydropyridines

In 10 mL of water, BF Co-Ag MOF@CuO nano-organocatalytic material (10 w/w percent, 0.018 g) was mixed with dimedone (2 mmol), aryl aldehyde (1 mmol), and NH_4_OAc (1 mmol) and heated to 60 °C for the necessary amount of time to undergo a chemical transformation. Using thin-layer chromatography (TLC) with a *n*-hexane: EtOAc (2:1) solvent mixture, the development of the chemical transformation was monitored. Following completion of the chemical transformation (as verified by TLC), 5 mL of hot 96% methanol was added, and the mixture was agitated for 2 min. Following filtration through Whatman filter paper (40), the catalytic material was extracted from the chemical transformation mixture and recovered for further use. The remaining solution was then poured over crushed ice, precipitating the product. The pure DHPs (4a-l) were obtained by recrystallization from ethanol (5 mL).

### spectral data

*3*,*3*,*6*,*6-tetramethyl-9-(3-nitrophenyl)−3*,*4*,*6*,*7*,*9*,*10-hexahydroacridine-1*,*8(2 H*,*5 H)-dione (****4a****)*: Yield 96%, m.p: 273–275 °C. ^1^H NMR (250 MHz, DMSO-*d*_*6*_): δ 0.83 (s, 3 H, CH_3_), 0.95 (s, 3 H, CH_3_), 1.03 (s, 3 H, CH_3_), 1.10 (s, 3 H, CH_3_), 2.23 (brs, 8 H, 4CH_2_), 4.36 (brs, 1H, CH), 6.08 (brs, 1H, NH), 7.50 (dd, 1H, *J*_1_= 6.37 Hz, *J*_2_= 14.5 Hz, H-Ar), 7.66 (t, 1H, J = 7 Hz, H-Ar), 7.78 (brs, 1H, H-Ar), 7.95 (brs, 1H, H-Ar) ppm; ^13^C NMR (62.5 MHZ, DMSO-*d*_*6*_): δ 26.8, 27.0, 28.2, 31.3, 31.8, 32.7, 33.0, 47.0, 49.1, 53.3, 57.4, 101.7, 120.7, 121.4, 123.2, 129.1, 129.9, 133.8, 135.5, 144.7, 148.2, 187.8, 196.3 ppm.

*3*,*3*,*6*,*6-tetramethyl-9-(m-tolyl)−3*,*4*,*6*,*7*,*9*,*10-hexahydroacridine-1*,*8(2 H*,*5 H)-dione (****4b****)*: Yield 95%, m, *p* = 257–258 °C. ^1^H NMR (250 MHz, DMSO-*d*_*6*_): δ 1.03 (s, 12 H, 4CH_3_), 2.19 (brs, 8 H, 4CH_2_), 2.31 (s, 3 H, CH_3_), 4.30 (s, 1H, CH), 5.87 (brs, 1H, NH), 6.77 (s, 1H, H-Ar), 6.84–7.06 (m, 3 H, H-Ar) ppm. ^13^C NMR (62.5 MHZ, DMSO-*d*_*6*_): δ 21.74, 26.96, 28.21, 31.25, 31.79, 32.66, 32.97, 46.97, 50.76, 53.28, 54.39, 100.69, 101.44, 115.00, 124.01, 126.17, 127.63, 128.20, 136.38, 137.06, 141.23, 187.78, 196.17 ppm.

*9-(2-chlorophenyl)−3*,*3*,*6*,*6-tetramethyl-3*,*4*,*6*,*7*,*9*,*10-hexahydroacridine-1*,*8(2 H*,*5 H)-dione (****4c****)*: Yield 96%, m.p: 216–218 °C. ^1^H NMR (250 MHz, DMSO-*d*_*6*_): δ 0.92 (s, 3 H, CH_3_), 0.97 (s, 3 H, CH_3_), 1.00 (s, 3 H, CH_3_), 1.07 (s, 3 H, CH_3_), 2.04–2.65 (m, 8 H, 4CH_2_), 4.55 (s, 1H, CH), 6.92–7.28 (m, 5 H, NH, 4 H-Ar) ppm. ^13^C NMR (62.5 MHZ, DMSO-*d*_*6*_): δ 26.8, 28.2, 29.3, 29.5, 31.7, 32.6, 33.1, 47.0, 49.1, 50.8, 55.3, 101.6, 109.4, 126.2, 126.6, 127.4, 129.0, 131.5, 132.5, 141.3, 167.9, 186.4, 196.1 ppm.

*3*,*3*,*6*,*6-tetramethyl-9-phenyl-3*,*4*,*6*,*7*,*9*,*10-hexahydroacridine-1*,*8(2 H*,*5 H)-dione (****4d****)*: Yield 93%, m, p: 285–287 °C. ^1^H NMR (250 MHz, DMSO-*d*_*6*_): δ 0.84 (s, 3 H, CH_3_), 1.02 (s, 6 H, 2CH_3_), 2.32 (brs, 8 H, 4CH_2_), 4.81 (s, 1H, CH), 5.94 (brs, 1H, NH), 6.95–7.15 (m, 5 H, H-Ar) ppm. ^13^C NMR (62.5 MHZ, DMSO-*d*_*6*_): δ 26.9, 28.2, 29.6, 31.3, 31.7, 32.6, 33.0, 47.0, 50.7, 53.3, 54.3, 111.9, 114.9, 125.5, 126.9, 128.0, 128.3, 129.0, 141.3, 147.6, 149.7, 187.8, 194.8 ppm.

*9-(4-chlorophenyl)−3*,*3*,*6*,*6-tetramethyl-3*,*4*,*6*,*7*,*9*,*10-hexahydroacridine-1*,*8(2 H*,*5 H)-dione (****4e****)*: Yield 95%, m, p: 227–230 °C. ^1^H NMR (250 MHz, DMSO-*d*_*6*_): δ 0.83 (s, 6 H, 2CH_3_), 0.99 (s, 6 H, 2CH_3_), 2.30 (brs, 8 H, 4CH_2_), 4.24 (s, 1H, CH), 5.92 (brs, 1H, NH), 6.84–7.23 (m, 4 H, H-Ar) ppm. ^13^C NMR (62.5 MHZ, DMSO-*d*_*6*_): δ 26.9, 28.2, 29.5, 30.9, 31.7, 32.7, 33.0, 47.0, 50.7, 53.3, 54.3, 101.5, 114.6, 127.5, 128.2, 128.7, 130.0, 130.2, 130.8, 140.8, 144.2, 187.6, 195.7 ppm.

*3*,*3*,*6*,*6-tetramethyl-9-(4-nitrophenyl)−3*,*4*,*6*,*7*,*9*,*10-hexahydroacridine-1*,*8(2 H*,*5 H)-dione (****4f****)*: Yield 98%, m, p: 280–282 °C. ^1^H NMR (250 MHz, DMSO-*d*_*6*_): δ 1.01 (s, 12 H, 4CH_3_), 2.32 (brs, 8 H, 4CH_2_), 4.35 (brs, 1H, CH), 6.08 (brs, 1H, NH), 7.31–7.43 (m, 2 H, H-Ar), 8.01–8.08 (m, 2 H, H-Ar) ppm. ^13^C NMR (62.5 MHZ, DMSO-*d*_*6*_): δ 26.9, 28.2, 31.8, 32.3, 32.8, 33.0, 33.7, 46.9, 50.3, 50.6, 53.2, 101.6, 114.3, 122.9, 123.6, 128.1, 129.6, 130.3, 145.7, 151.1, 153.8, 187.6, 196.5 ppm.

*9-(4-methoxyphenyl)−3*,*3*,*6*,*6-tetramethyl-3*,*4*,*6*,*7*,*9*,*10-hexahydroacridine-1*,*8(2 H*,*5 H)-dione (****4 g****)*: Yield 98%, m, p: 273–274 °C. ^1^H NMR (250 MHz, DMSO-*d*_*6*_): δ 0.83 (s, 3 H, CH_3_), 0.95 (s, 3 H, CH_3_), 1.03 (s, 3 H, CH_3_), 1.10 (s, 3 H, CH_3_), 2.34 (brs, 8 H, 4CH_2_), 4.36 (s, 1H, CH), 6.08 (brs, 1H, NH), 7.41–7.78 (m, 3 H, H-Ar), 7.95 (s, 1H, H-Ar) ppm. ^13^C NMR (62.5 MHZ, DMSO-*d*_*6*_): δ 26.8, 28.2, 29.0, 31.3, 31.8, 32.7, 33.0, 47.0, 50.6, 53.3, 57.4, 113.8, 120.6, 121.4, 123.2, 129.1, 129.9, 133.8, 144.6, 147.5, 148.2, 187.8, 196.6 ppm.

*9-(2*,*4-dichlorophenyl)−3*,*3*,*6*,*6-tetramethyl-3*,*4*,*6*,*7*,*9*,*10-hexahydroacridine-1*,*8(2 H*,*5 H)-dione (****4 h****)*: Yield 97%, m, p: 317–320 °C. ^1^H NMR (400 MHz, DMSO-*d*_*6*_): δ 0.48 (s, 3 H, CH_3_), 0.72 (s, 3 H, CH_3_), 0.84 (s, 3 H, CH_3_), 0.93 (s, 3 H, CH_3_), 1.39 (brs, 1H, NH), 1.66 (dd, 2 H, *J*_*1*_= 6.4 Hz, *J*_*2*_= 53.2 Hz, CH_2_), 2.17–2.47 (m, 6 H, 3CH_2_), 5.04 (s, 1H, CH), 7.03–7.05 (m, 2 H, H-Ar), 7.15 (t, 1H, *J* = 7.4 Hz, H-Ar), 7.22–7.26 (m, 1H, H-Ar), 7.49 (d, *J* = 8.8 Hz, 2 H, H-Ar), 7.55 (d, *J* = 7.6 Hz, 1H, H-Ar), 10.46 (brs, 1H, OH) ppm. ^13^C NMR (100 MHZ, DMSO-*d*_*6*_): δ 26.8, 28.2, 29.0, 31.3, 31.8, 32.7, 33.0, 47.0, 50.6, 53.3, 57.4, 113.8, 120.6, 121.4, 123.2, 129.1, 129.9, 133.8, 144.6, 147.5, 148.2, 187.8, 196.6 ppm.

*9-(2-methoxyphenyl)−3*,*3*,*6*,*6-tetramethyl-3*,*4*,*6*,*7*,*9*,*10-hexahydroacridine-1*,*8(2 H*,*5 H)-dione (****4i****)*: Yield 98%, m, p: 297–298 °C. ^1^H NMR (250 MHz, DMSO-*d*_*6*_): δ 0.90 (s, 3 H, CH_3_), 0.95 (s, 3 H, CH_3_), 1.00 (s, 3 H, CH_3_), 1.05 (s, 3 H, CH_3_), 2.04 (brs, 2 H, CH_2_), 2.11 (brs, 2 H, CH_2_), 2.22–2.48 (m, 4 H, 2CH_2_), 4.49 (s, 1H, CH), 7.01 (d, *J* = 2 Hz, 1H, H-Ar), 7.07 (d, *J* = 2 Hz, 1H, H-Ar), 7.14 (t, *J* = 2 Hz, 1H, H-Ar), 7.45 (s, 1H, NH) ppm. ^13^C NMR (62.5 MHZ, DMSO-*d*_*6*_): δ 26.6, 26.7, 28.2, 29.0, 29.5, 32.6, 33.1, 47.1, 49.0, 50.7, 55.0, 101.6, 109.1, 114.0, 126.4, 128.3, 131.0, 132.8, 133.3, 140.5, 168.2, 186.2, 196.1 ppm.

*9-(3-chlorophenyl)−3*,*3*,*6*,*6-tetramethyl-3*,*4*,*6*,*7*,*9*,*10-hexahydroacridine-1*,*8(2 H*,*5 H)-dione (****4j****)*: Yield 95%, m.p: 195–197 °C. ^1^H NMR (250 MHz, DMSO-*d*_*6*_, ppm): δ 0.83 (s, 3 H, CH_3_), 0.95 (s, 3 H, CH_3_), 1.03 (s, 3 H, CH_3_), 1.10 (s, 3 H, CH_3_), 2.34 (brs, 8 H, 4CH_2_), 4.36 (s, 1H, CH), 6.08 (brs, 1H, NH), 7.41–7.78 (m, 3 H, H-Ar), 7.95 (s, 1H, H-Ar) ppm. ^13^C NMR (62.5 MHZ, DMSO-*d*_*6*_): δ 26.8, 28.2, 29.0, 31.3, 31.8, 32.7, 33.0, 47.0, 50.6, 53.3, 57.4, 113.8, 120.6, 121.4, 123.2, 129.1, 129.9, 133.8, 144.6, 147.5, 148.2, 187.8, 196.6 ppm.

*9-(2-hydroxynaphthalen-1-yl)−3*,*3*,*6*,*6-tetramethyl-3*,*4*,*6*,*7*,*9*,*10-hexahydroacridine-1*,*8(2 H*,*5 H)-dione (****4k****)*: Yield 95%, m, p: 175–177 °C. ^1^H NMR (400 MHz, DMSO-*d*_*6*_): δ 0.48 (s, 3 H, CH_3_), 0.72 (s, 3 H, CH_3_), 0.84 (s, 3 H, CH_3_), 0.93 (s, 3 H, CH_3_), 1.39 (brs, 1H, NH), 1.66 (dd, 2 H, *J*_*1*_= 6.4 Hz, *J*_*2*_= 53.2 Hz, CH_2_), 2.17–2.47 (m, 6 H, 3CH_2_), 5.04 (s, 1H, CH), 7.03–7.05 (m, 2 H, H-Ar), 7.15 (t, 1H, *J* = 7.4 Hz, H-Ar), 7.22–7.26 (m, 1H, H-Ar), 7.49 (d, *J* = 8.8 Hz, 2 H, H-Ar), 7.55 (d, *J* = 7.6 Hz, 1H, H-Ar), 10.46 (brs, 1H, OH) ppm. ^13^C NMR (100 MHZ, DMSO-*d*_*6*_): δ 26.8, 28.2, 29.0, 31.3, 31.8, 32.7, 33.0, 47.0, 50.6, 53.3, 57.4, 113.8, 120.6, 121.4, 123.2, 129.1, 129.9, 133.8, 144.6, 147.5, 148.2, 187.8, 196.6 ppm.

*9-(2*,*4-dimethoxyphenyl)−3*,*3*,*6*,*6-tetramethyl-3*,*4*,*6*,*7*,*9*,*10-hexahydroacridine-1*,*8(2 H*,*5 H)-dione (****4 L****)*: Yield 99%, m, p: 198–201 °C. ^1^H NMR (250 MHz, DMSO-d_6_): δ 0.81 (s, 3 H, CH_3_), 0.90 (s, 3 H, CH_3_), 0.96 (s, 3 H, CH_3_), 1.06 (s, 3 H, CH_3_), 1.97–2.48 (m, 8 H, 4CH_2_), 3.66 (s, 3 H, OCH_3_), 3.68 (s, 3 H, OCH_3_), 4.39 (s, 1H, CH), 6.26–6.74 (m, 3 H, H-Ar), 9.15 (brs, 1H, NH) ppm. ^13^C NMR (62.5 MHZ, DMSO-d_6_): δ 27.0, 28.0, 29.3, 31.6, 32.4, 32.6, 33.0, 47.0, 49.2, 50.9, 53.2, 55.4, 55.7, 97.9, 101.6, 104.0, 110.2, 115.5, 123.8, 129.8, 157.2, 158.6, 167.2, 186.4, 196.0 ppm.

## Results and discussion

### Characterization and Synthesis of BF Co-Ag MOF@CuO nanorods

**Fig. 2:**
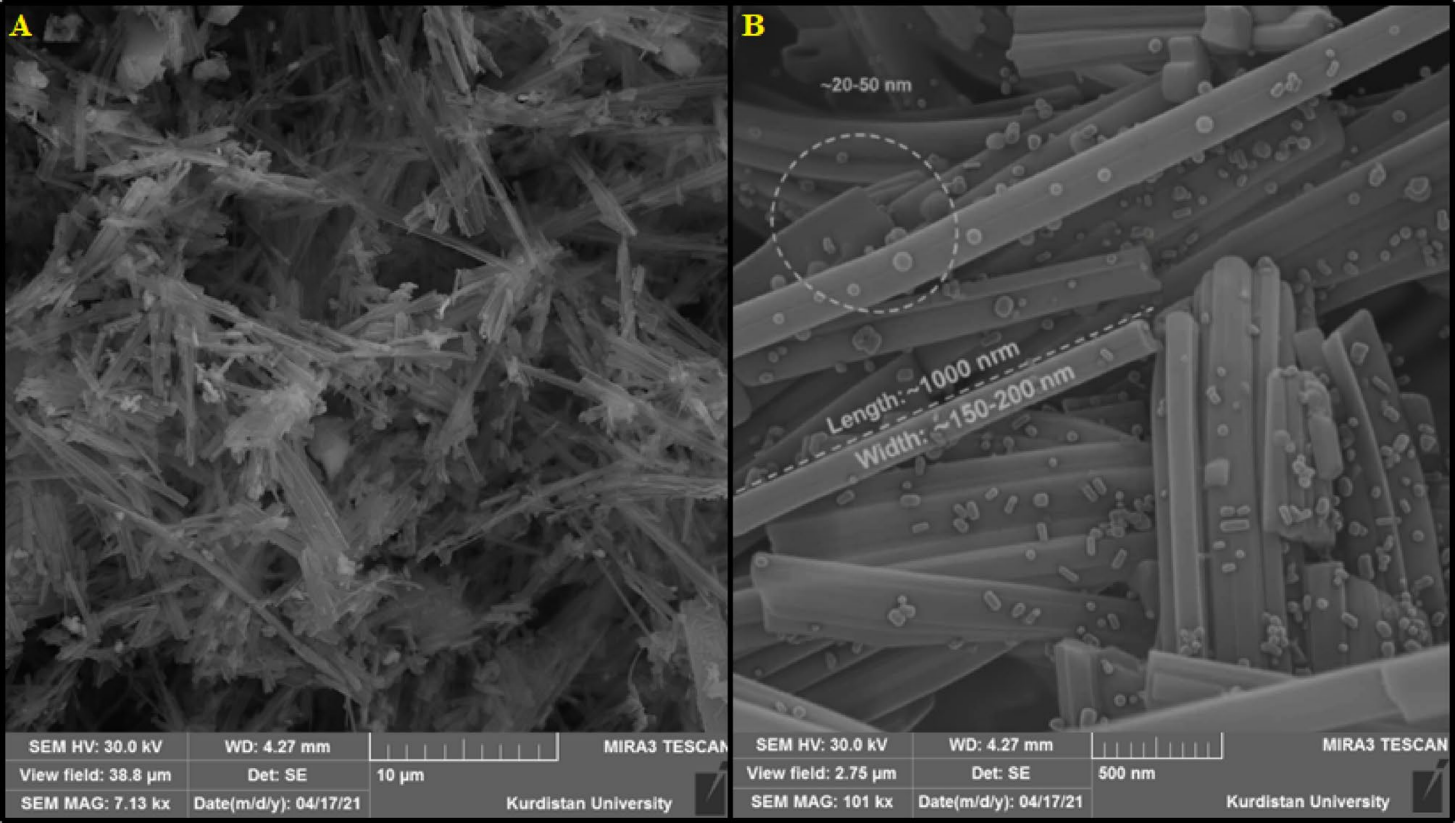
(**a**) SEM image and (**b**) high-resolution SEM image of BF Co-Ag MOF@CuO nanorods.

The morphological features and structural integrity of the synthesized BF Co-Ag MOF@CuO nanocomposite were investigated using FE-SEM and TEM. The FE-SEM micrographs reveal a distinct rod-like morphology for the BF Co-Ag MOF (Fig. 2(a)). These nanorods exhibit high structural uniformity with an average length of approximately 1–3 *µ*m and a width ranging between 150 and 200 nm (Fig. 2(b)). A key observation is the successful “decoration” of the nanorod surfaces with smaller spherical entities. These CuO nanoparticles (NPs), with a diameter of roughly 20–50 nm, are distributed across the MOF nanorods (Fig. 2(b)). The high surface-to-volume ratio of the nanorods, combined with the uniform distribution of CuO NPs, suggests an abundance of accessible active sites, which is critical for enhanced catalytic performance.

**Fig. 3:**
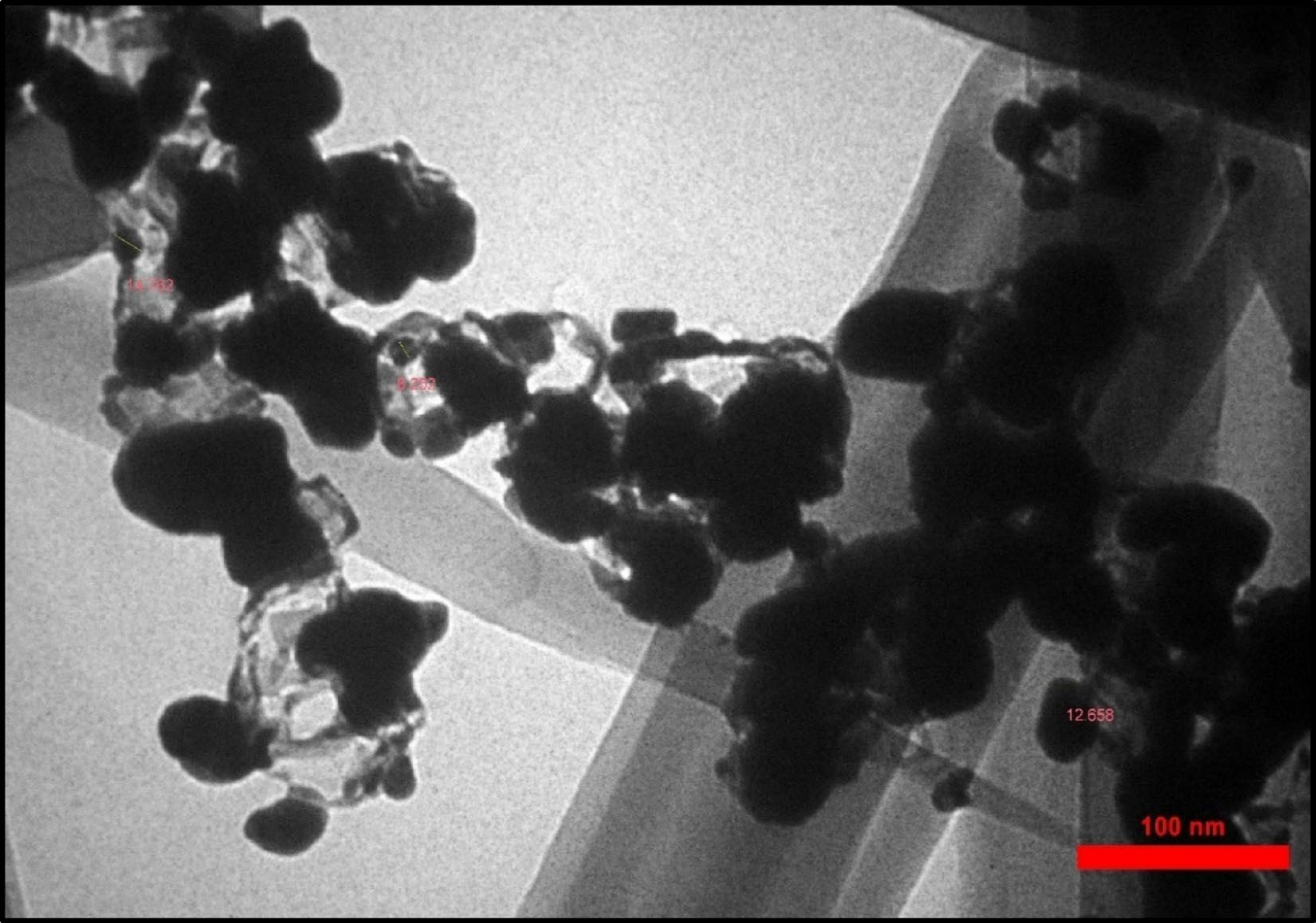
TEM image of CuO NPs stabilized on BF Co-Ag MOF substrate.

Figure 3 depicts the TEM image of BF Co-Ag MOF@CuO nanocomposite. The TEM images confirm the hybrid nature of the composite, showing the high-contrast (darker) CuO nanoparticles firmly anchored onto the lower-contrast (lighter) Co-Ag MOF nanorods. The calcination process appears to play a crucial role in producing samples with stable morphology. Based on this image, the successful fabrication of BF Co-Ag MOF@CuO nanocomposite material or one-dimensional nanostructures is confirmed.

**Fig. 4:**
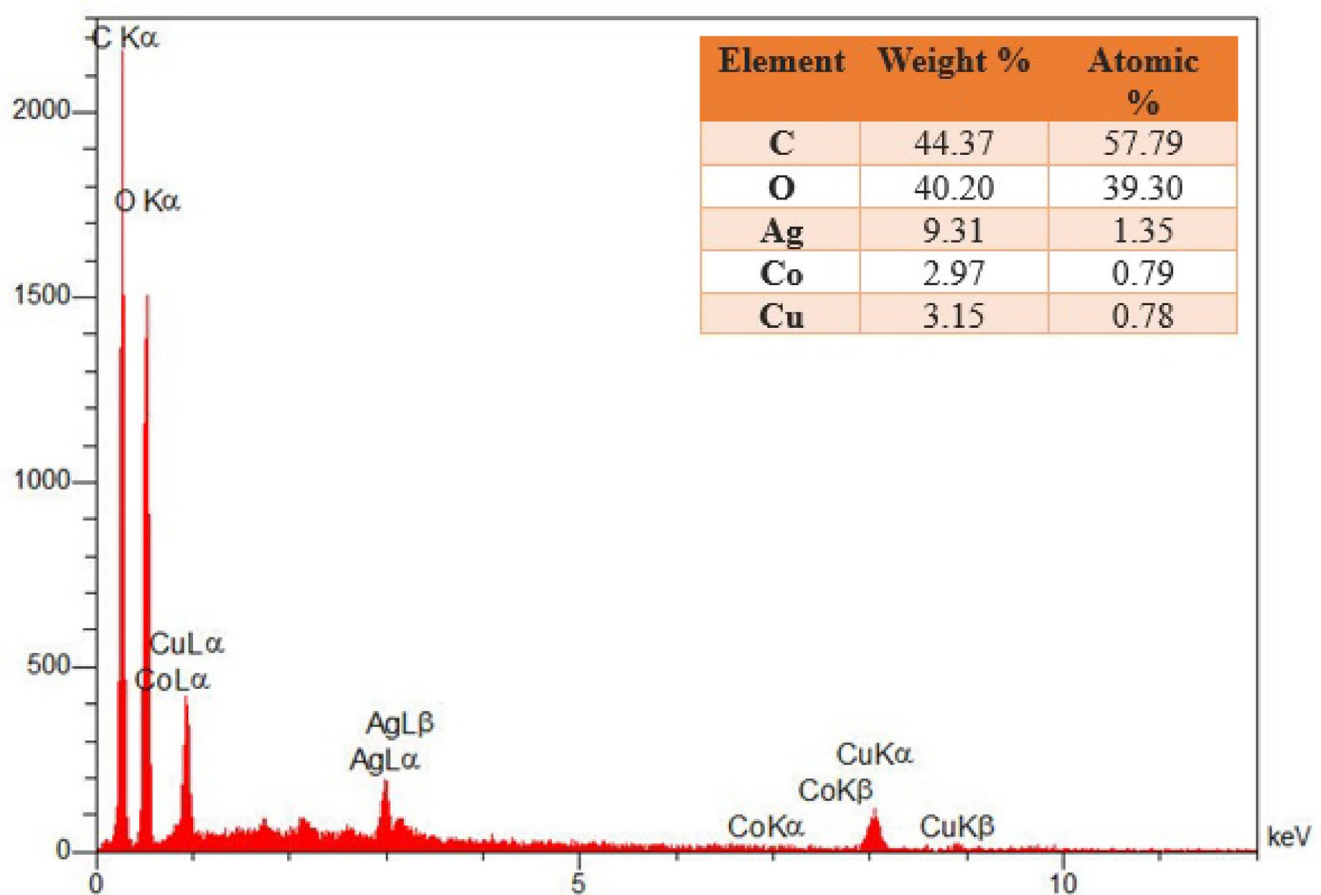
EDX spectra of BF Co-Ag MOF@CuO nanorods.

Figure 4 presents the EDX analysis of BF Co-Ag MOF@CuO nanorods, revealing the presence of C, O, N, Cu, Co, and Ag. The quantitative analysis confirms the successful integration of CuO nanoparticles onto the BF Co-Ag MOF. The results (inset in Fig. 4) reveal a high carbon and oxygen content, which is attributed to the trimesic acid (TMA) organic linkers. The host framework consists of Co(II) and Ag(I) ions coordinated with the TMA linkers. The EDX data indicate a higher weight% of Ag (9.31 W%) compared to Co (2.97 W%), which is consistent with the higher molar mass of Ag relative to Co despite the competitive coordination during the precipitation process. The presence of Cu (3.15 W%) confirms the successful loading of CuO nanoparticles.

**Fig. 5:**
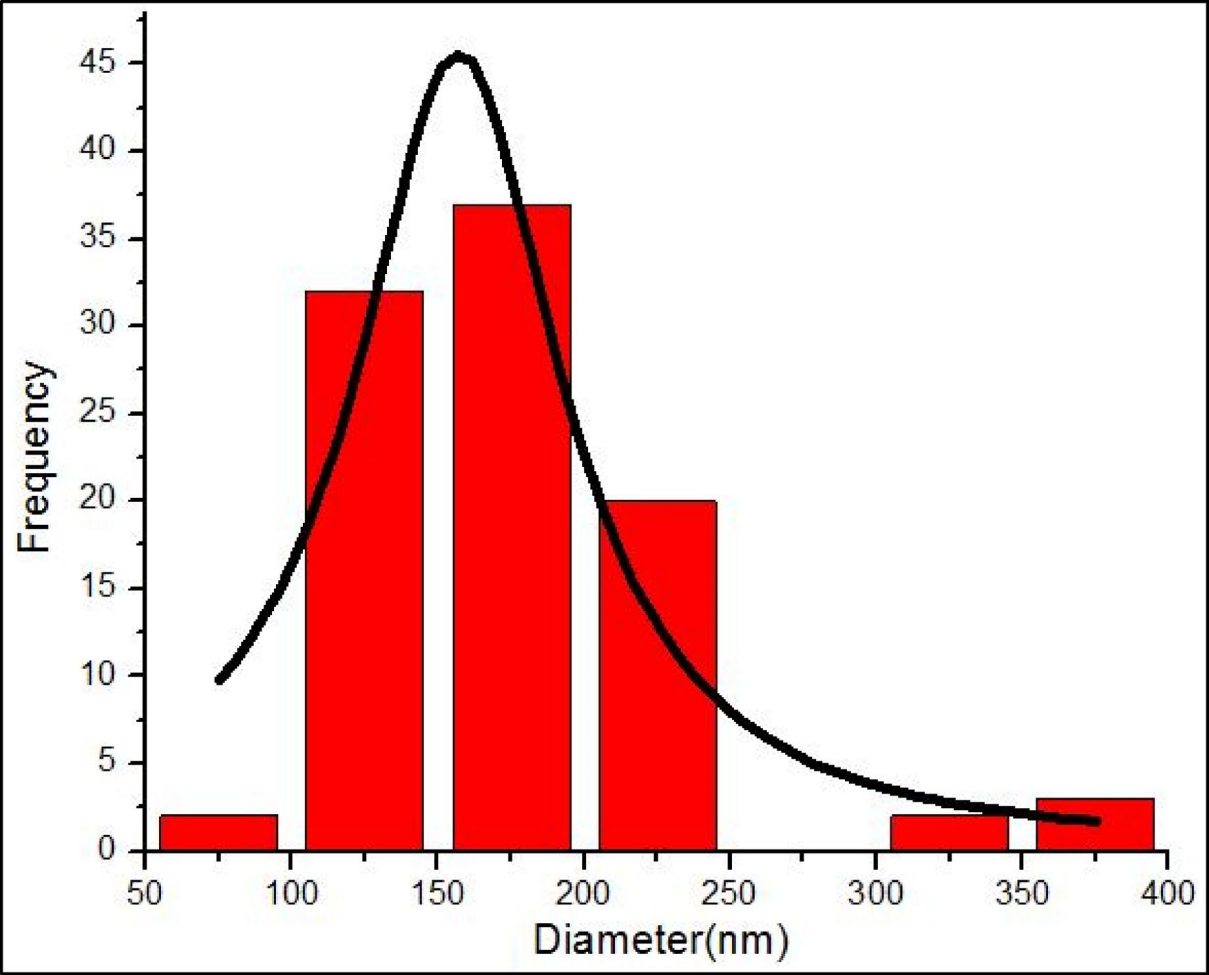
Histogram of particle size distribution from FE-SEM images of BF-Co-Ag MOF@CuO nanocatalyst.

Figure 5 presents the histogram of particle size distribution of Co-Ag-MOF@CuO nanostructures. According to this analysis, the particle size distribution is homogeneous, which aligns with the SEM and TEM results. The uniform distribution of catalytic material particles provides optimal conditions for catalytic processes, achieving maximum efficiency within a selective range.

**Fig. 6:**
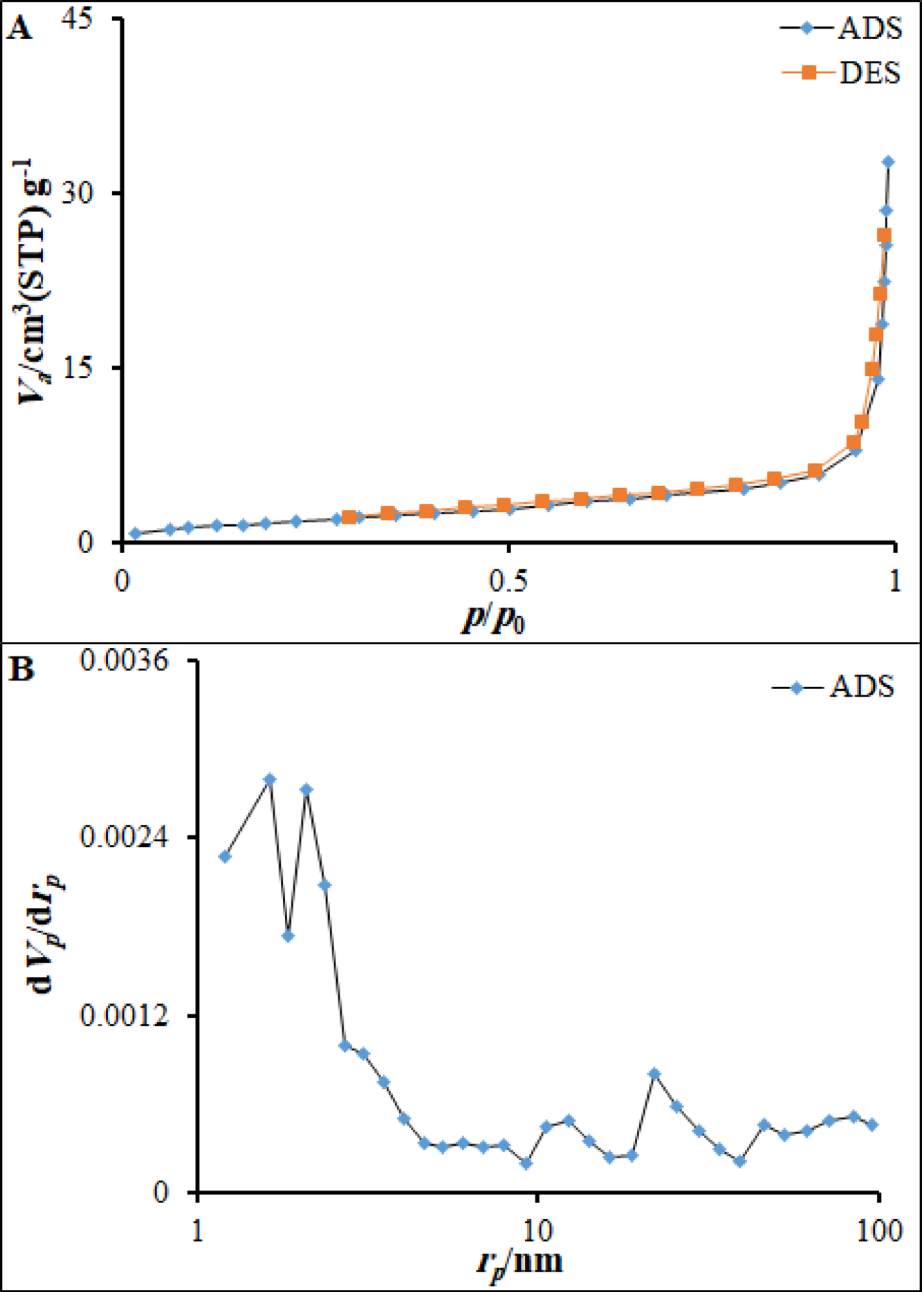
(**a**) N_2_ adsorption-desorption isotherms of BF Co-Ag MOF@ CuO nanorods and (**b**) BJH results obtained for BF Co-Ag MOF@ CuO nanorods. ^a^ ADS: Adsorption, ^b^ DES: Desorption.

The N_2_ adsorption-desorption isotherm of BF Co-Ag MOF@CuO nanorods is shown in Fig. 6. To evaluate important physical characteristics like surface area, pore size distribution, and pore volume, N_2_ sorption analysis was performed. The isotherm is consistent with a Type IV pattern, characterized by a clear hysteresis loop. The nanorods’ measured pore sizes, pore volumes, and surface areas are 26.474 nm, 0.046 cm^3^/g, and 7.0337 m^2^/g, respectively.

**Fig. 7:**
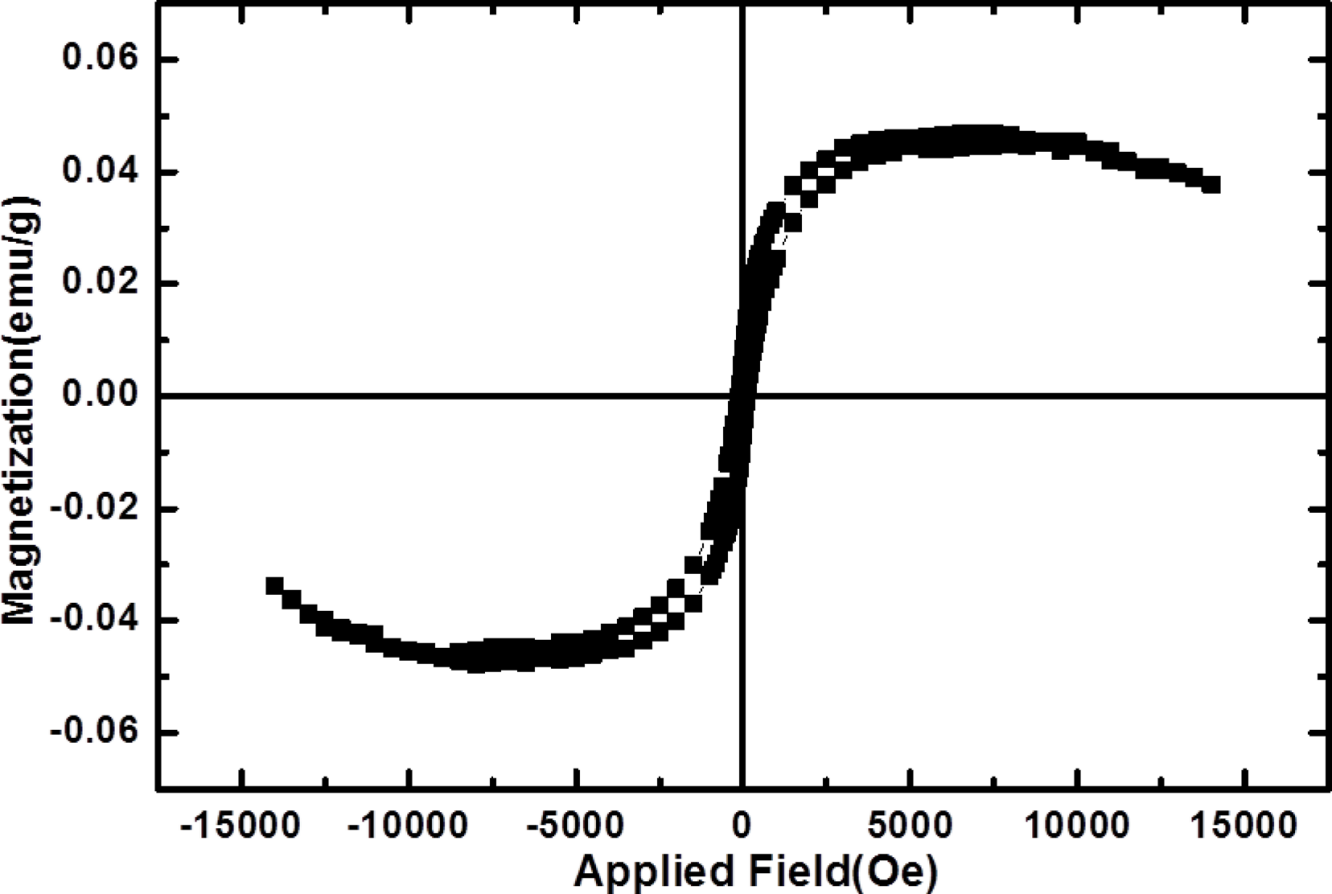
VSM magnetization curves of BF Co-Ag MOF@CuO nanorods.

The magnetic properties of the BF Co-Ag MOF@CuO nanorods were evaluated using a VSM at room temperature. Figure 7 displays the M-H curve of the nanocomposite under these conditions. The results clearly indicate that the magnetic behavior of the semiconductor materials is closely influenced by their structure, morphology, and crystal geometry. The M-H curve is typical of a weak paramagnetic material, as the spin orientation is restricted at the maximum applied field of 0.052 Oe. The nanocomposite exhibits a coercivity (Hc) of 100.0 Oe, which reflects its magnetic behavior, and demonstrates saturation magnetism (Ms) of 0.012 emu·g⁻¹.

**Fig. 8:**
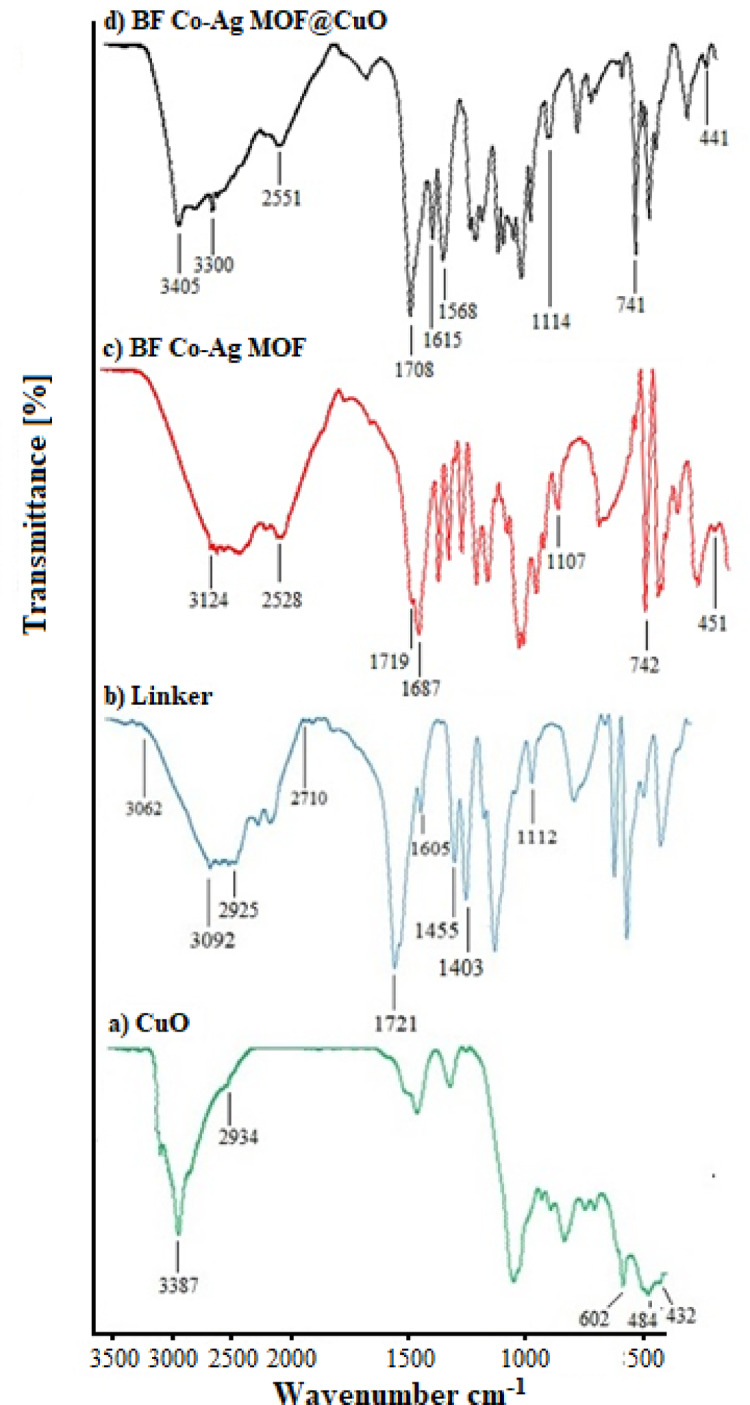
IR (KBr, υ/cm^− 1^) curve of the (**a**) CuO, (**b**) trimesic acid, (**c**) synthesized BF Co-Ag MOF and (**d**) BF Co-Ag MOF@CuO nanorods.

As catalytic materials, the FT-IR spectrum confirms the existence of functional groups in organic compounds and the creation of new functional groups in the BF Co-Ag MOF@CuO nanorods and synthesized MOFs (Fig. 8a, b, c, and d). The carbonyl groups at about 1721 cm^− 1^ and the C–O single stretching bonds at about 1455 and 1403 cm^− 1^ are important functional groups in the benzene-1,3,5-tricarboxylic acid (Trimesic acid (TMA)) ligand^56–58^.

The broad band observed at 2528–3124 cm^− 1^ in Co-Ag MOF is attributed to the stretching vibration of hydroxyl groups. The absorption peak around 451 cm^− 1^ corresponds to the metal–oxygen–hydrogen bending vibration (Co–OH) in this system. Additionally, the peaks at 1107 and 742 cm^− 1^ are associated with the C–O–Co bond in Co-BTC. As shown in Fig. 8c, the C = O bond peak splits into two peaks at 1719 and 1687 cm^− 1^ for TMA-Co-Ag MOF, indicating the interaction between Co and Ag ions and carboxylate groups. This interaction shifts the C = O stretching frequency from 1721 cm^− 1^ (characteristic of TMA) to higher wavelengths and lower energy levels at 1687 and 1719 cm^− 1^.

Based on the IR spectra results, it can be concluded that the BF Co-Ag MOF@CuO nanorods were successfully synthesized. In the spectrum of the TMA ligand, the characteristic peaks for –OH and C = O were observed at 3092, 1721, and 1605 cm^− 1^ (blue curve). However, upon coordination with Ag⁺ and Co^2+^, these peaks shifted to 3300, 1615, and 1568 cm^− 1^ (black curve).

### Preparation of fused 1,4-dihydropyridines via BF Co-Ag MOF@CuO nanorods as nano-organo catalytic material

**Fig. 9:**
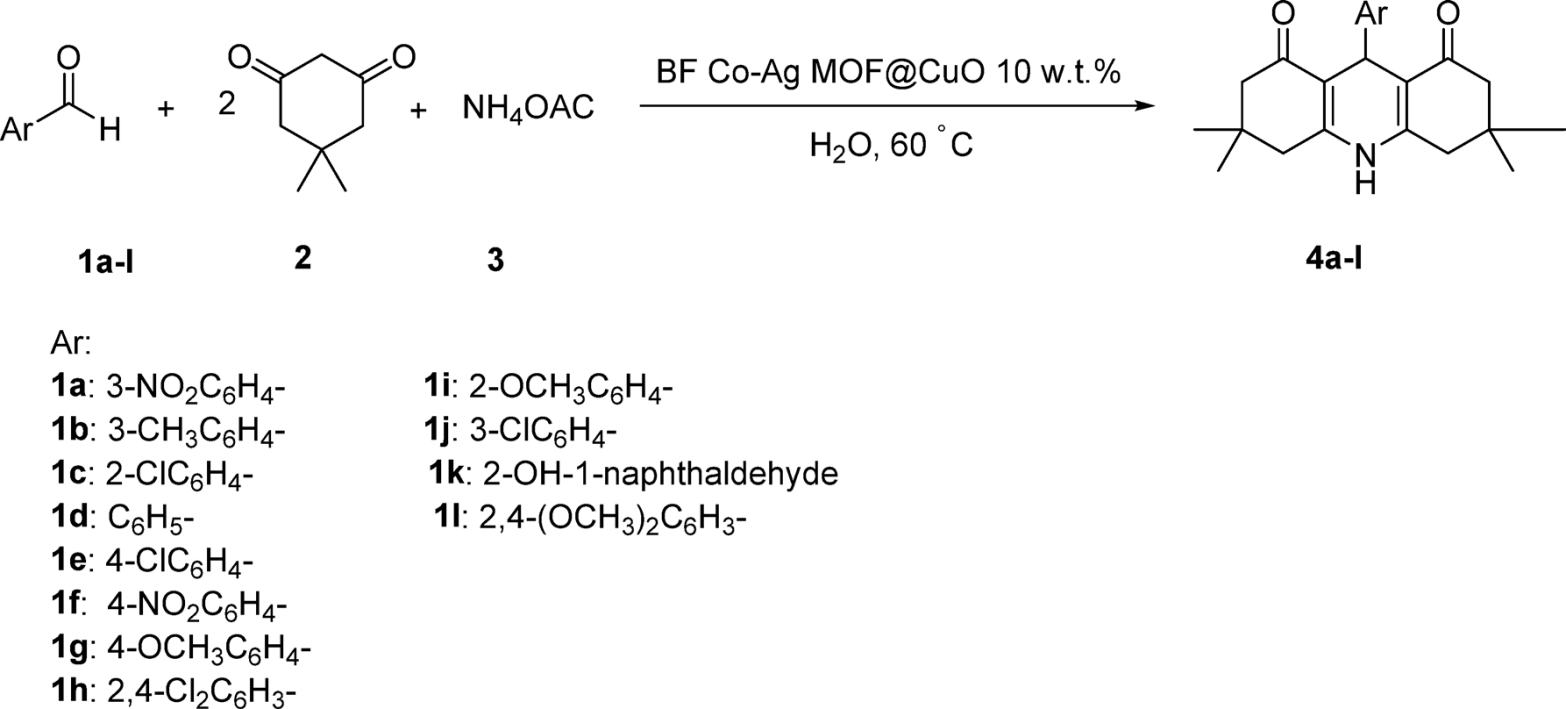
Preparation of 1, 4-dihydropyridines.

As part of our ongoing efforts to develop a highly effective and environmentally friendly method for synthesizing pharmaceutical compounds^49–54^, and considering the significant biological and pharmaceutical properties of DHPs, we investigated the catalytic potential and activity of the newly synthesized BF Co-Ag MOF@CuO nanorods. After preparing and characterizing the catalytic material, its efficiency was evaluated in the modified Hantzsch chemical transformation, where dimedone was reacted with various aromatic aldehydes (1a-l) and NH_4_OAc. The optimal chemical transformation conditions were determined by examining different parameters, including solvent choice, catalyst loading, and temperature, using a model reaction involving 3-nitrobenzaldehyde (3 mmol), dimedone (6 mmol), and NH_4_OAc (6 mmol) (Fig. 9).

At the beginning of the investigation, the influence of various solvents, including H_2_O, EtOH, MeOH, EtOH: H_2_O, MeOH: H_2_O, (CH_3_)_2_CO, DMF, CH_3_CN, and CH_2_Cl_2_, was examined in a model chemical transformation of 4-chlorobenzaldehyde, dimedone and ammonium acetate using 5 w.t.% BF Co-Ag MOF@CuO nanorods catalytic material under reflux conditions and also, solvent free conditions in 100 °C. After careful investigation, it was observed that the chemical transformation proceeded effectively in all tested solvents; however, H_2_O was identified as the optimal choice, yielding the highest yield (Table 1, entry 10). In industrial chemistry, water is considered an ideal solvent due to its green nature, low cost, and wide availability, making it a preferred medium for such chemical transformations.

Following solvent optimization, we looked into how temperature affected the model chemical transformation. Even after 7 h of stirring, nothing was produced at room temperature; only the raw ingredients remained. We gradually increased the temperature from room temperature to 90 °C to provide additional thermal energy. The findings demonstrated that the yield of the target product rose in direct proportion to temperature, peaking at 60 °C (Table 1, Entry 12).

To evaluate the impact of the catalytic material and determine the optimal catalytic material loading for maximum yield, the model chemical transformation was conducted both without a catalytic material and with varying amounts (5, 10, 15, and 20 w.t.%) of BF Co-Ag MOF@CuO nanorods (Table 1). The findings showed that even after a prolonged chemical transformation period of seven hours, only a trace amount of the intended product was produced in the absence of a catalytic material (Table 1, Entry 14). Furthermore, since 10 w.t.% was sufficient to drive the chemical transformation to completion, increasing the amount of catalytic material beyond that level had no discernible impact on the yield or the time of the reaction (Table 1, Entry 15).

Therefore, the ideal chemical transformation conditions were established as 10 w.t.% of the catalytic material in an aqueous medium at 60 °C.

**Table 1:** Optimization of the chemical transformation conditions for the fabrication of fused 1,4-dihydropyridines using BF Co-Ag MOF@CuO nanorods. ^a^Reaction conditions: 4-chlorobenzaldehyde (3 mmol), dimedone (6 mmol), and ammonium acetate (6 mmol) in presence of BF Co-Ag MOF@CuO under different conditions. ^b^Yield refer to isolated products. By reacting different aromatic aldehydes with dimedone and ammonium acetate, the scope and general applicability of this procedure were investigated, thanks to the exceptional optimization results obtained (Table 2). Aldehydes with electron-donating and electron-withdrawing groups participated in the condensation chemical transformation with equal effectiveness, it was discovered. Thus, the chemical transformation was not significantly affected by the kind and location of substituents on the aromatic ring. By contrasting the products’ melting points, they were identified. Furthermore, ^1^H and^13^ C NMR spectra were used to confirm the structures of every compound.

Entry ^a^	Catalyst	Solvent	Temperature (^0^C)	Time (min/h)	Yield (%) ^b^
1	BF Co-Ag MOF@CuO nanorods 5 w.t.%	Free-solvent	100 °C	3–4 min	61
2	BF Co-Ag MOF@CuO nanorods 5 w.t.%	CH_3_OH	reflux	10 min	70
3	BF Co-Ag MOF@CuO nanorods 5 w.t.%	C_2_H_5_OH	reflux	4 min	62
4	BF Co-Ag MOF@CuO nanorods 5 w.t.%	CH_3_CN	reflux	4 min	57
5	BF Co-Ag MOF@CuO nanorods 5 w.t.%	C_3_H_6_O	reflux	8 min	64
6	BF Co-Ag MOF@CuO nanorods 5 w.t.%	DMF	reflux	8 min	51
7	BF Co-Ag MOF@CuO nanorods 5 w.t.%	CH_2_Cl_2_	reflux	5 min	59
8	BF Co-Ag MOF@CuO nanorods 5 w.t.%	C_2_H_5_OH: H_2_O	reflux	5 min	65
9	BF Co-Ag MOF@CuO nanorods 5 w.t.%	CH_3_OH: H_2_O	reflux	6 min	75
10	BF Co-Ag MOF@CuO nanorods 5 w.t.%	H_2_O	reflux	3 min	81
11	BF Co-Ag MOF@CuO nanorods 5 w.t.%	H_2_O	r.t.	7 h	----
12	BF Co-Ag MOF@CuO nanorods 5 w.t.%	H_2_O	60 °C	2–3 min	88
13	BF Co-Ag MOF@CuO nanorods 5 w.t.%	H_2_O	90 °C	2–3 min	88
14	Free- catalyst	H_2_O	r.t.	7 h	Trace
15	BF Co-Ag MOF@CuO nanorods 10 w.t.%	H_2_O	60 °C	2–3 min	98
16	BF Co-Ag MOF@CuO nanorods 15 w.t%	H_2_O	60 °C	2–3 min	98
17	BF Co-Ag MOF@CuO nanorods 20 w.t%	H_2_O	60 °C	2–3 min	98

**Table 2:** Synthesis of fused 1,4-dihydropyridine derivatives (4a–l) using 10 w.t.% of BF Co-Ag MOF@CuO nanocatalytic material in an aqueous medium at 60 °C. ^a^ Reaction conditions: Aldehyde (3 mmol), dimedone (6 mmol), and ammonium acetate (6 mmol) in the presence of BF Co-Ag MOF@CuO (10 w.t.%) in H_2_O at temperature of 60 °C. ^b^ Isolated yields after purification.

Entry	*R* (aldehyde)	Product	Time (min)	Yield (%)^b^	$\frac{{m.p.\left( {{}^ \circ C} \right)}}{{FoundReported[ref.]}}$
1	3-NO_2_C_6_H_4_-	4a	3	96	273–275 272–274^59^
2	3-CH_3_C_6_H_4_-	4b	4	95	257–258 253–255^59^
3	2-ClC_6_H_4_-	4c	3	96	216–218 218–220^60^
4	C_6_H_5_-	4d			285–287 286–288^59^
5	4-ClC_6_H_4_-	4e	5	95	227–230 228–230^59^
6	4-NO_2_C_6_H_4_-	4f	10	98	280–282 281–282^60^
7	4-OCH_3_C_6_H_4_-	4 g	2	98	273–274 272–274^59^
8	2,4-Cl_2_C_6_H_3_-	4 h	3	97	317–320 > 321^43^
9	2-OCH_3_C_6_H_4_-	4i	2	98	297–298 296–298^61^
10	3-ClC_6_H_4_-	4j			195–197 New
11	2-OHC_10_H_6_-	4k	5	95	175–177 New
12	2,4-(OCH_3_)_2_C_6_H_3_-	4 L	2	99	198–201 New

**Fig. 10:**
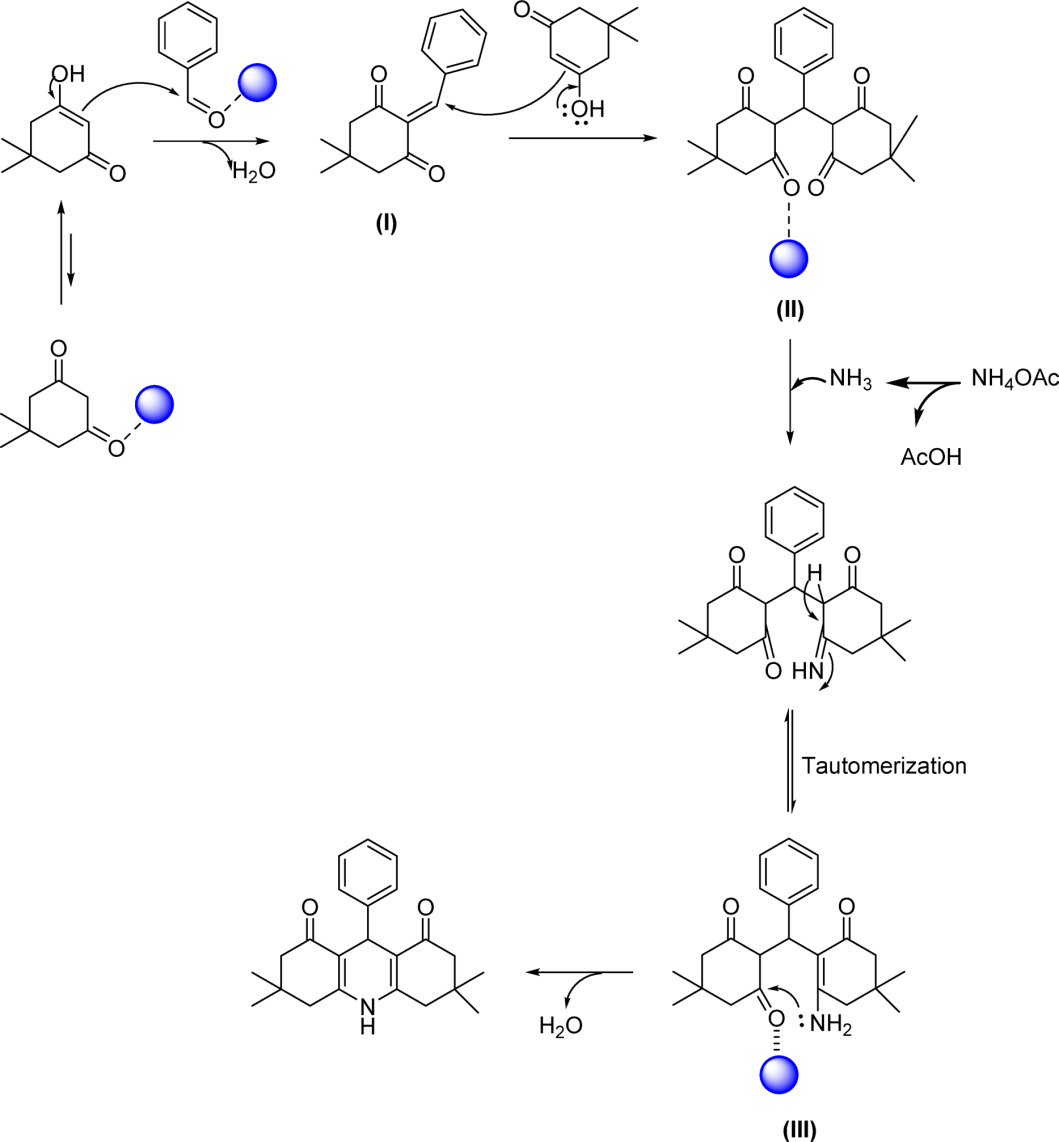
Proposed mechanism for preparation of 1,4-dihydropyridine derivatives.

A plausible mechanism for the fabrication of 1,4-DHPs using the BF Co-Ag MOF@CuO nano-catalytic material is illustrated in Fig. 10. Initially, an acid-base interaction occurs between the nano-catalytic material and the oxygen in the carbonyl bonds of both the aryl aldehyde and dimedone. The nano-catalytic material binds to the oxygen atoms of the carbonyl groups, forming covalent bonds between the carbonyl groups of the aryl aldehyde and dimedone. As a result, the BF Co-Ag MOF@CuO catalytic material activates the aryl aldehyde while simultaneously increasing the reactivity of the dimedone carbonyl, making its alpha hydrogen highly acidic. This promotes enolization and makes it easier for the aryl aldehyde to be attacked by a nucleophile, leading to a Knoevenagel condensation and the formation of intermediate (I). To create intermediate (II), intermediate (I) is subjected to a Michael addition with an enolized dimedone molecule. Following hydrolysis, this intermediate combines with ammonia liberated from ammonium acetate to form an enamine (III). In the last stage, the amine group attacks the carbonyl carbon nucleophilically, leading to intramolecular cyclization of the enamine. Dehydration comes next, which finally yields the intended product^62^.

To demonstrate the characteristics and efficiency of the proposed method, we performed a comparison between the efficiency of the synthesized catalytic material and some catalytic materials used in other proposed methods by previous researchers for the chemical transformation of 4-chlorobenzaldehyde, dimedone, and ammonium acetate (4a) based on product yield and chemical transformation completion time (Table 3).

**Table 3:** Comparison of the catalytic activity efficiency of BF Co-Ag MOF@CuO nanorods as nano-organocatalytic materials with catalytic materials reported in previous articles for the preparation of fused 1,4-dihydropyridines.

Entry	Catalyst	Amount of catalyst	Conditions	Time (min/h)	Yield (%)	Ref
1	Preyssler heteropolyacid	0.01 g	H_2_O, reflux	180 min	85	60
2	salicylic acid (0.2 equiv.)	0.2 equiv.	Solvent-free, 80 °C	3 h	82	63
3	nano-FGT	0.02 g	Solvent-free, 110 °C	30 min	91	59
4	glycerol	5 mL	65 °C	68 min	96	62
5	CdS thin film NPs	1 g	EtOH, 75 °C	2 h	88	64
6	Fe_3_O_4_@SiO_2_ -SnCl_4_	25 mg	EtOH, reflux	25 min	91	11
7	Cell-Pr-NHSO_3_H	0.05 g, 0.0076 mmol	EtOH, reflux	40 min	92	65
8	L-proline	15 mol%	H_2_O, reflux	180 min	85	66
9	Nano- Fe_3_O_4_	0.05 g	H_2_O, reflux	10 min	96	67
10	Fe_3_O_4_@SiO_2_ NPs	10 mol%	H_2_O, reflux	20 min	92	13
11	HY-zeolite	0.1 g	EtOH, reflux	2.5 h	75	68
12	CBSA	0.03 g	Solvent-free, 100 °C	20 min	93	69
13	BF Co-Ag MOF@CuO nanorods	10 w.t.%	H_2_O, 60 °C	5 min	95	This work

Beyond the process’s outstanding outcomes, the BF Co-Ag MOF@CuO nano-catalytic material’s ease of product isolation and recyclability greatly increase the effectiveness of the chemical transformation. This offers organic fabrication a significant benefit. We tried to repurpose the catalytic material under the same chemical transformation conditions to make the process more economically and environmentally feasible.

**Fig. 11:**
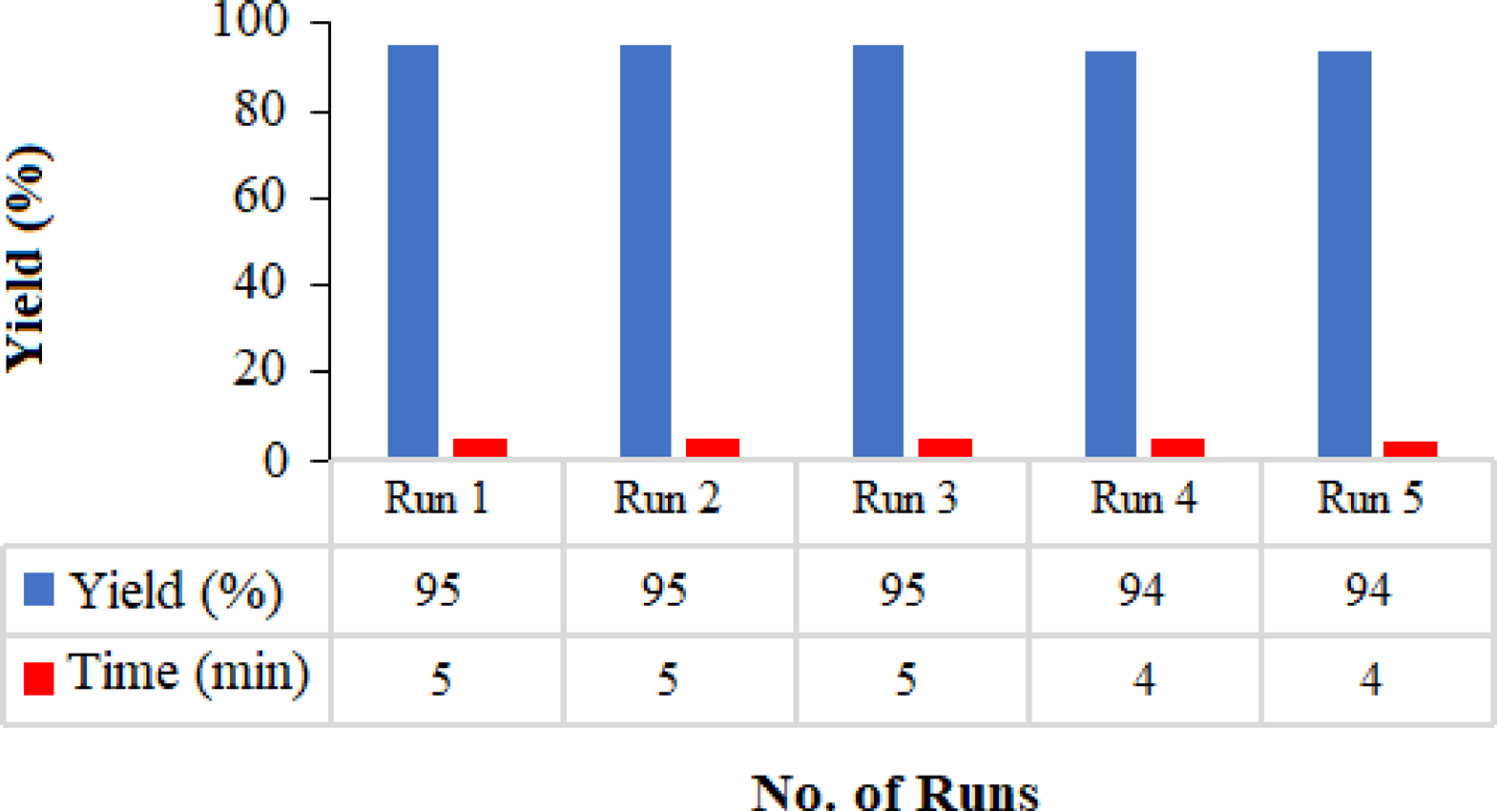
BF Co-Ag MOF@CuO nano-catalytic material recovery diagram in the preparation of 3,3,6,6-tetramethyl-9-(3-nitrophenyl)−3,4,6,7,9,10-hexahydroacridine-1,8(2 H,5 H)-dione (**4a**) compound.

It should be noted that the catalytic material can be recycled up to 5 times, yielding good product yields (Fig. 11) with negligible activity loss. We examined the catalytic material’s high catalytic activity even after the fifth reuse to determine whether it would become poisoned and lose catalytic efficacy during the chemical transformation. By fabricating compound 4a, the recovery and reusability of the BF Co-Ag MOF@CuO nanocatalytic material were investigated. Following the completion of the chemical transformation, the nano-catalytic material was recovered by dissolving it in methanol, separated using Whatman filter paper (40), and then rinsed with 3 × 10 mL of water and 3 × 10 mL of methanol before being dried for 6 h at 50 °C in an air oven. Before using the catalytic material again in the subsequent chemical transformation under the same circumstances, no additional purification was required. Because the catalytic material is not sensitive to moisture or air, it does not need to be handled or stored in any special way (Fig. 11).

**Fig. 12:**
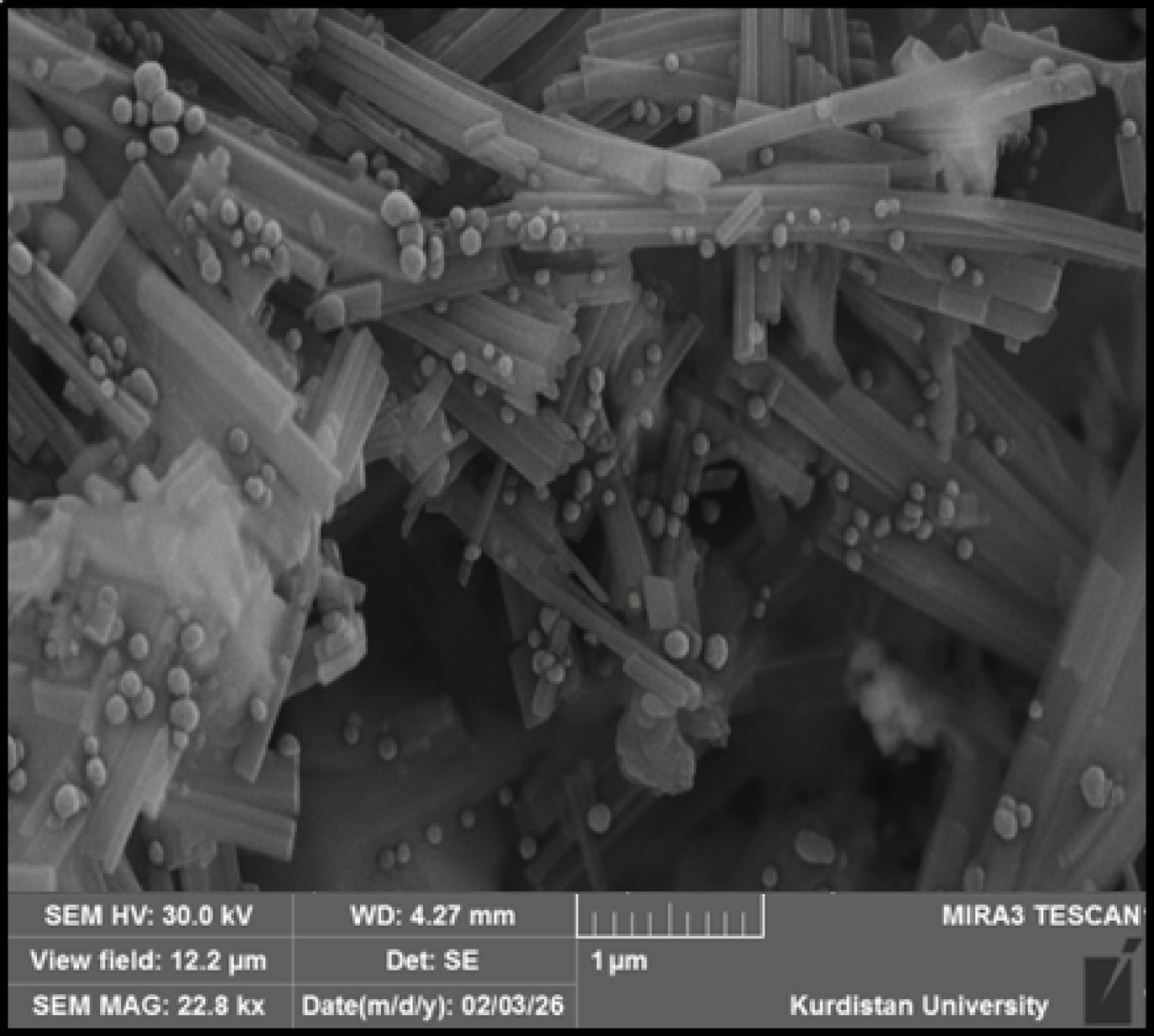
SEM image of BF Co-Ag MOF@CuO nano-catalytic material after the recycling procedure.

SEM, EDX, and X-ray analyses were used to characterize the recovered Ag_2_O NP@IOP nanocatalytic material further. Figure 12 shows the SEM image of the nanocatalytic material after regeneration. The results indicate a rod-shaped morphology, demonstrating that the morphology remains unchanged after the catalytic regeneration process. A comparison with the pre-regeneration image shows no significant alterations in particle size distribution, confirming that the organic compounds have no impact on particle size during the catalytic process. Moreover, no evidence of structural aggregation was observed in the final product, validating the composite’s stability. The high surface stability of the nanocatalytic material suggests its potential for reuse in multiple catalytic cycles.

Figure S-25 presents the EDX analysis of the catalytic material after regeneration. As is evident, the constituent elements of the catalytic material are well-retained in the final structure following the catalytic process. This finding provides strong evidence for the structural integrity of the catalytic material during functional operation. A detailed comparison with the initial EDS analysis in Fig. 4 reveals that the elemental composition of the initial catalytic material is preserved in the final structure.

Figure S-26 shows the X-ray diffraction (XRD) pattern of the nanocatalytic material after the regeneration process. Based on this pattern, the crystalline structure of the final product is well preserved. Since one of the key features of MOFs is their high crystallinity, this observation strongly confirms the presence of MOF in the final structure. Upon closer examination, minor noise in certain regions of the pattern indicates the presence of residual organic compounds that were not catalyzed in the final structure.

## Conclusion

For the first time, a novel, highly effective heterogeneous catalytic material was prepared and reported in this study. Using pertinent analytical techniques, the catalytic material’s physicochemical characteristics were characterized, including a narrow particle size distribution, uniform morphology, and significant porosity. Additionally, by reacting various aryl aldehydes, dimedone, and ammonium acetate in aqueous media at 60 °C, the BF Co-Ag MOF@CuO nanorods’ suitability for synthesizing different fused 1,4-dihydropyridine derivatives was assessed. Short chemical transformation times, a straightforward execution and workup process, substantial product outputs, and a chemical that produces no waste are just a few benefits of the developed procedure.

## Supplementary Information

Below is the link to the electronic supplementary material.

Supplementary Material 1

## Data Availability

The original contributions presented in the study are included in the article/Supplementary material; further inquiries can be directed to the corresponding author.
