# Supplementary material for: Synthesis and characterization of bi-functional Co-Ag MOF@CuO nanorods as an innovative robust heterogeneous catalytic material for the fabrication of fused 1,4-dihydropyridine derivatives
Source: Sci Rep. 2026 May 18;16:22582. doi: 10.1038/s41598-026-43843-8 (PMC13381576; doi:10.1038/s41598-026-43843-8)
Supplement: Supplementary file 1 — Supplementary Material 1 [file 41598_2026_43843_MOESM1_ESM.docx]

***Supplementary Material***

**Synthesis and characterization of bi-functional Co-Ag MOF@CuO** **nanorods as an innovative robust heterogeneous catalytic material for the fabrication of fused** **1,4-dihydropyridine derivatives**

**Negar Hoot ^1^, Enayatollah Sheikhhosseini ^1^*, Sayed Ali Ahmadi ^1^, Mahdieh Yahyazadehfar^1^**

**Department of Chemistry, Ke.C., Islamic Azad University, Kerman, Iran.**

* **Correspondence:**

Enayatollah Sheikhhosseini

sheikhhosseiny@gmail.com or e.sheikhhosseini@iau.ir

**^1^H NMR and ^13^C NMR of compound (4a)**

*3,3,6,6-tetramethyl-9-(3-nitrophenyl)-3,4,6,7,9,10-hexahydroacridine-1,8(2H,5H)-dione (****4a****)*: Yield: 96%, M.p. = 273-275 ^°^C. ^1^H NMR (250 MHz, DMSO-*d_6_*, ppm) δ: 0.83 (s, 3H, CH_3_), 0.95 (s, 3H, CH_3_), 1.03 (s, 3H, CH_3_), 1.10 (s, 3H, CH_3_), 2.23 (brs, 8H, 4CH_2_), 4.36 (brs, 1H, CH), 6.08 (brs, 1H, NH), 7.50 (dd, 1H, *J_1_*= 6.37 Hz, *J_2_*= 14.5 Hz, H-Ar), 7.66 (t, 1H, *J* = 7 Hz, H-Ar), 7.78 (brs, 1H, H-Ar), 7.95 (brs, 1H, H-Ar). ^13^C NMR (62.5 MHZ, DMSO-*d_6_*, ppm) δ: 26.8, 27.0, 28.2, 31.3, 31.8, 32.7, 33.0, 47.0, 49.1, 53.3, 57.4, 101.7, 120.7, 121.4, 123.2, 129.1, 129.9, 133.8, 135.5, 144.7, 148.2, 187.8, 196.3.

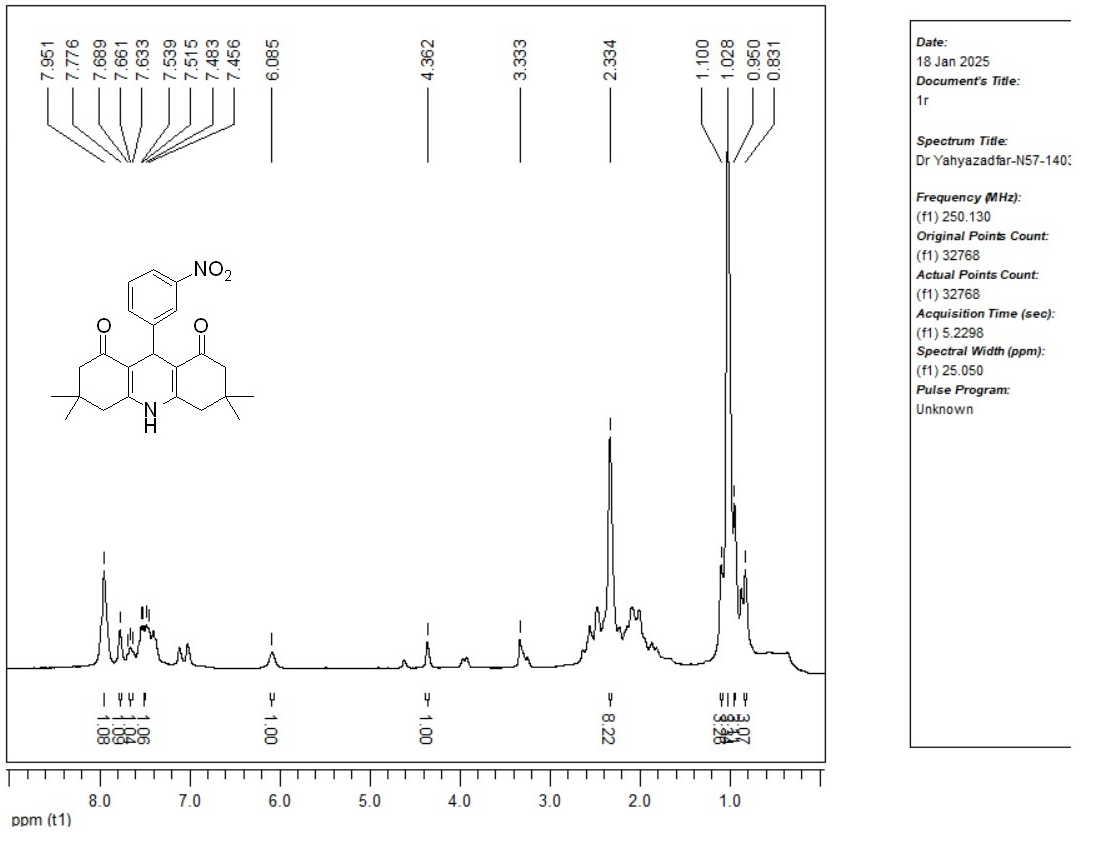


**Figure S-1.** ^[1](https://www.google.com/url?sa=t&rct=j&q=&esrc=s&source=web&cd=&cad=rja&uact=8&ved=2ahUKEwjmjuPvw579AhW_8LsIHTvtAd8QFnoECBAQAQ&url=https%3A%2F%2Fwww.researchgate.net%2Ffigure%2FThe-1-H-NMR-spectrum-of-compound-3a-in-CDCl-3-solvent_fig1_320865085&usg=AOvVaw2S68h8geITZVHy7tcJjbIy)^[H NMR spectrum of compound 4a.](https://www.google.com/url?sa=t&rct=j&q=&esrc=s&source=web&cd=&cad=rja&uact=8&ved=2ahUKEwjmjuPvw579AhW_8LsIHTvtAd8QFnoECBAQAQ&url=https%3A%2F%2Fwww.researchgate.net%2Ffigure%2FThe-1-H-NMR-spectrum-of-compound-3a-in-CDCl-3-solvent_fig1_320865085&usg=AOvVaw2S68h8geITZVHy7tcJjbIy)


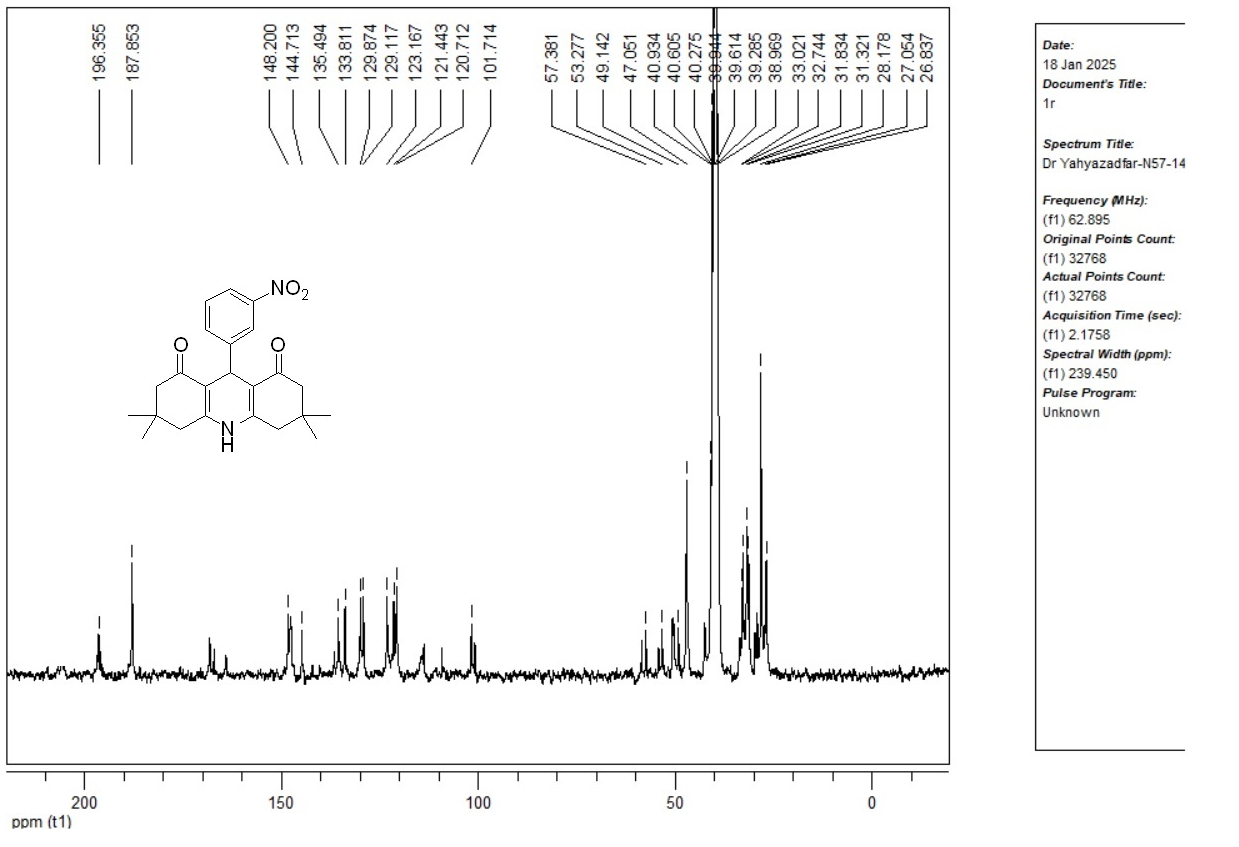


**Figure S-2.** ^13^C NMR spectrum of compound 4a.

**^1^H NMR and ^13^C NMR of compound (4b)**

*3,3,6,6-tetramethyl-9-(m-tolyl)-3,4,6,7,9,10-hexahydroacridine-1,8(2H,5H)-dione (****4b****):* Yield: 95%, m,p. = 257-258 ^°^C. ^1^H NMR (250 MHz, DMSO-*d_6_*, ppm) δ: 1.03 (s, 12H, 4CH_3_), 2.19 (brs, 8H, 4CH_2_), 2.31 (s, 3H, CH_3_), 4.30 (s, 1H, CH), 5.87 (brs, 1H, NH), 6.77 (s, 1H, H-Ar), 6.84-7.06 (m, 3H, H-Ar). ^13^C NMR (62.5 MHZ, DMSO-*d_6_*, ppm) δ: 21.74, 26.96, 28.21, 31.25, 31.79, 32.66, 32.97, 46.97, 50.76, 53.28, 54.39, 100.69, 101.44, 115.00, 124.01, 126.17, 127.63, 128.20, 136.38, 137.06, 141.23, 187.78, 196.17.

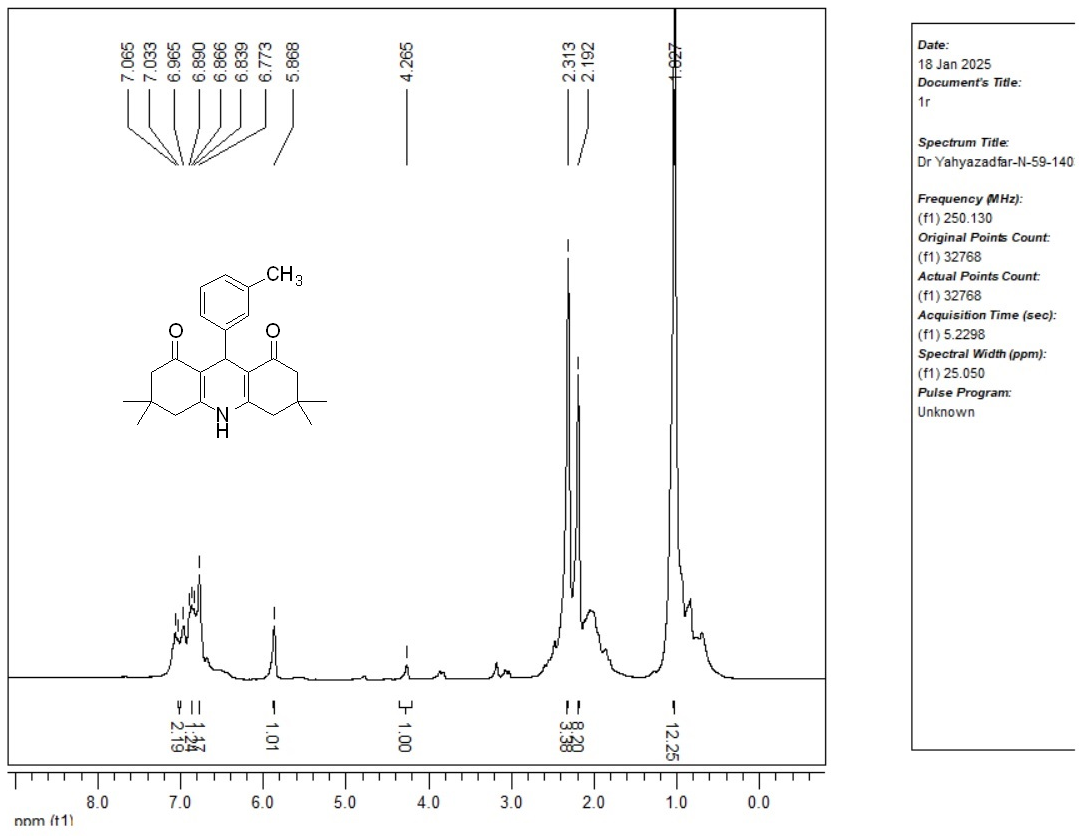


**Figure S-3.** ^1^H NMR expand spectrum of compound 4b.


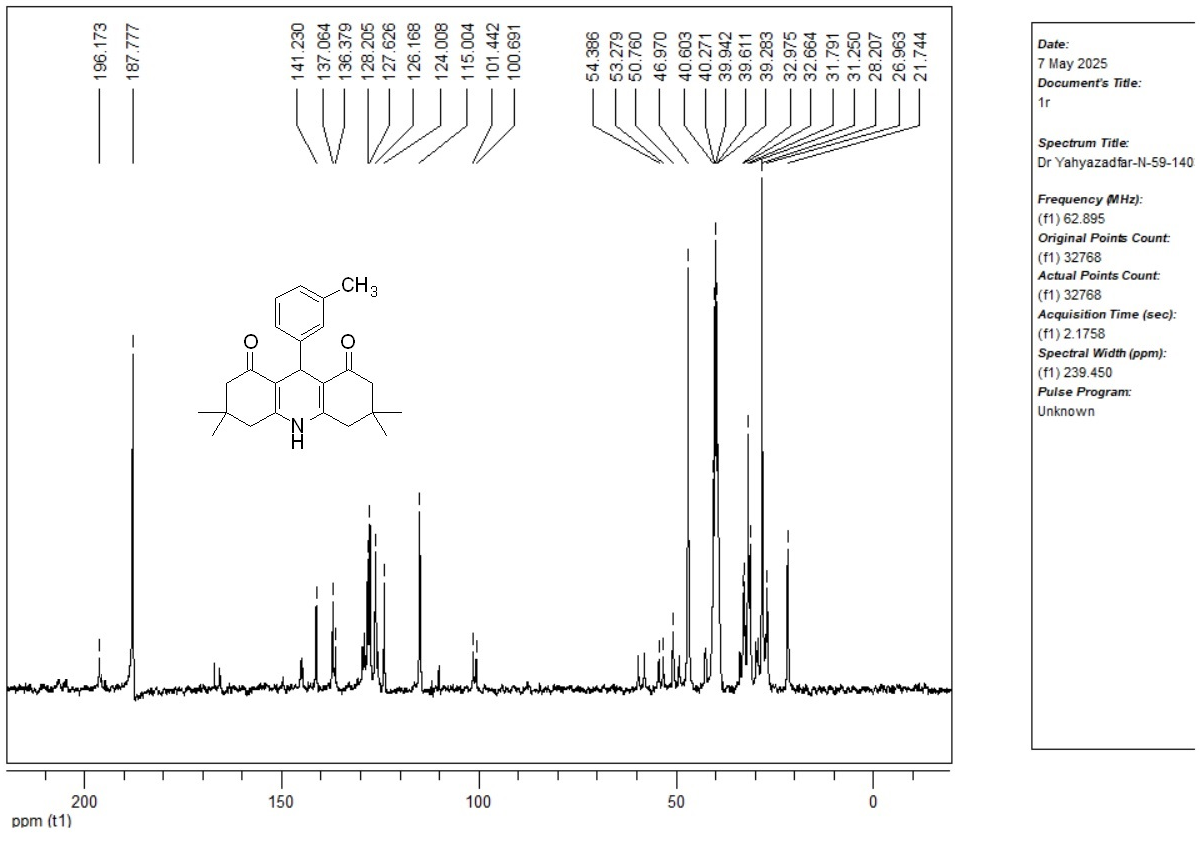


**Figure S-4.** ^13^C NMR spectrum of compound 4b.

**^1^H NMR and ^13^C NMR of compound (4c)**

*9-(2-chlorophenyl)-3,3,6,6-tetramethyl-3,4,6,7,9,10-hexahydroacridine-1,8(2H,5H)-dione (****4c****)*: Yield: 96%, m,p. = 216-218 ^°^C. ^1^H NMR (250 MHz, DMSO-*d_6_*, ppm) δ: 0.92 (s, 3H, CH_3_), 0.97 (s, 3H, CH_3_), 1.00 (s, 3H, CH_3_), 1.07 (s, 3H, CH_3_), 2.04-2.65 (m, 8H, 4CH_2_), 4.55 (s, 1H, CH), 6.92-7.28 (m, 5H, NH, 4H-Ar). ^13^C NMR (62.5 MHZ, DMSO-*d_6_*, ppm) δ: 26.8, 28.2, 29.3, 29.5, 31.7, 32.6, 33.1, 47.0, 49.1, 50.8, 55.3, 101.6, 109.4, 126.2, 126.6, 127.4, 129.0, 131.5, 132.5, 141.3, 167.9, 186.4, 196.1.

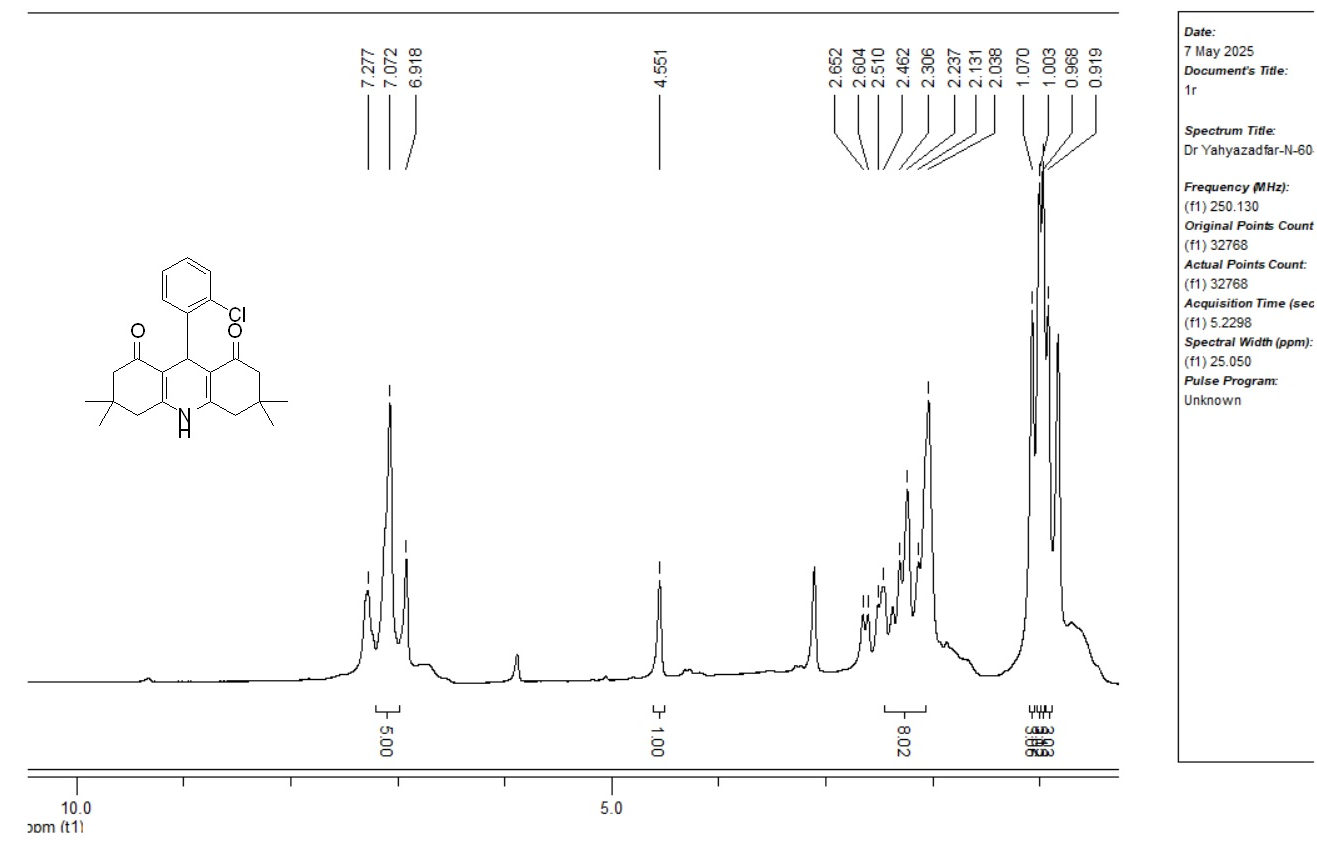


**Figure S-5.** ^1^H NMR spectrum of compound 4c.


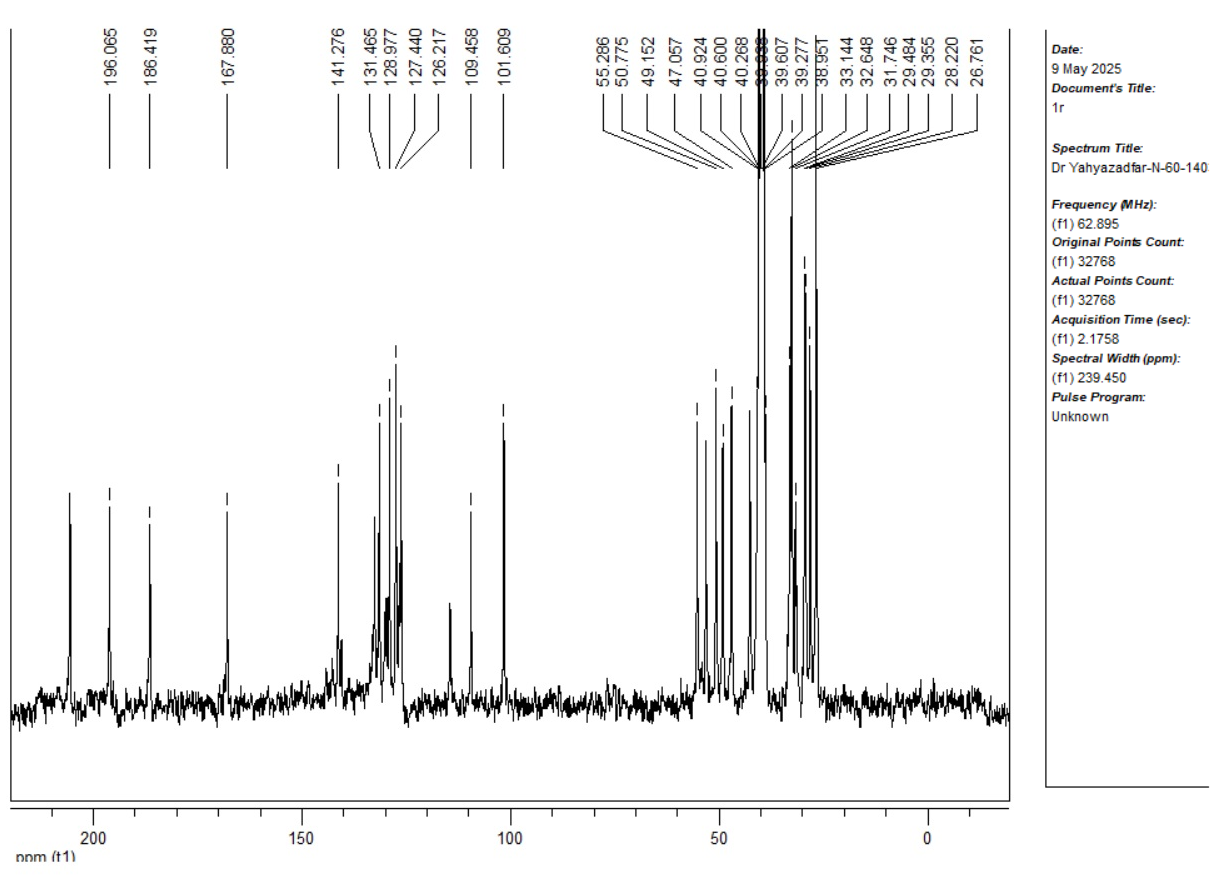


**Figure S-6.** ^13^C NMR spectrum of compound 4c.

**^1^H NMR and ^13^C NMR of compound (4d)**

*3,3,6,6-tetramethyl-9-phenyl-3,4,6,7,9,10-hexahydroacridine-1,8(2H,5H)-dione (****4d****)*: Yield: 93%, m,p. = 285-287 ^°^C. ^1^H NMR (250 MHz, DMSO-*d_6_*, ppm) δ: 0.84 (s, 3H, CH_3_), 1.02 (s, 6H, 2CH_3_), 2.32 (brs, 8H, 4CH_2_), 4.81 (s, 1H, CH), 5.94 (brs, 1H, NH), 6.95-7.15 (m, 5H, H-Ar). ^13^C NMR (62.5 MHZ, DMSO-*d_6_*, ppm) δ: 26.9, 28.2, 29.6, 31.3, 31.7, 32.6, 33.0, 47.0, 50.7, 53.3, 54.3, 111.9, 114.9, 125.5, 126.9, 128.0, 128.3, 129.0, 141.3, 147.6, 149.7, 187.8, 194.8.

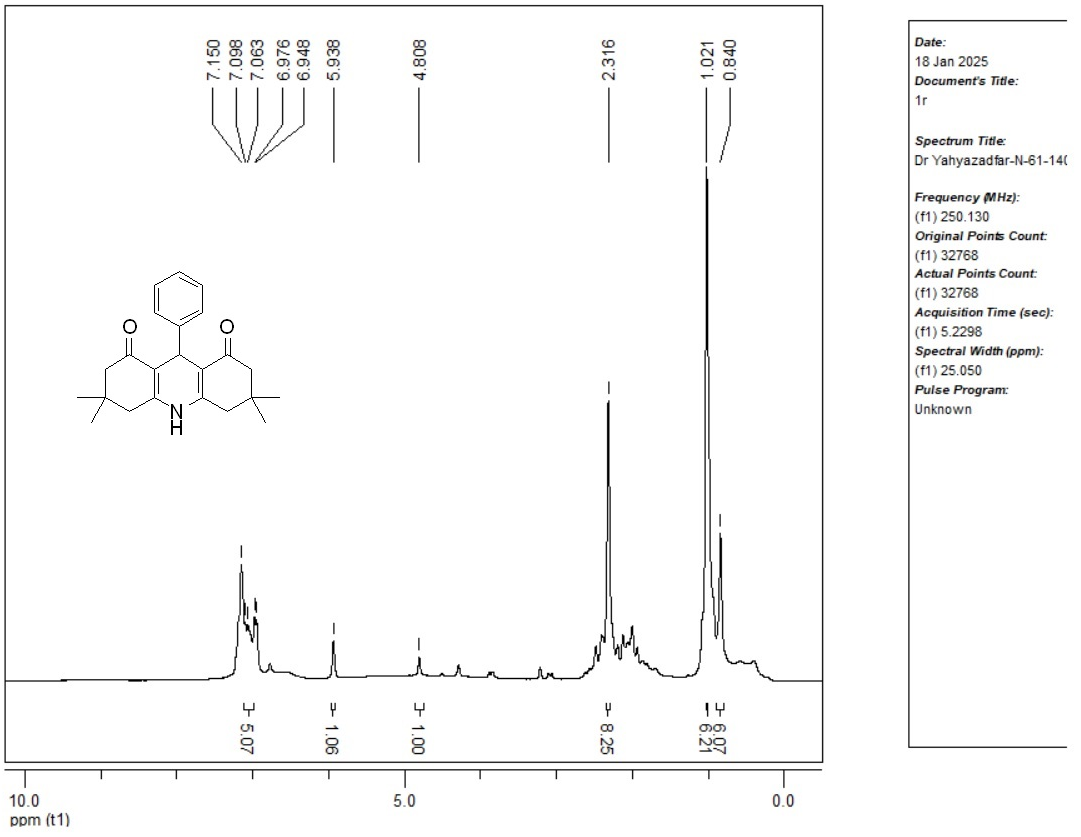


**Figure S-7.** ^1^H NMR spectrum of compound 4d.


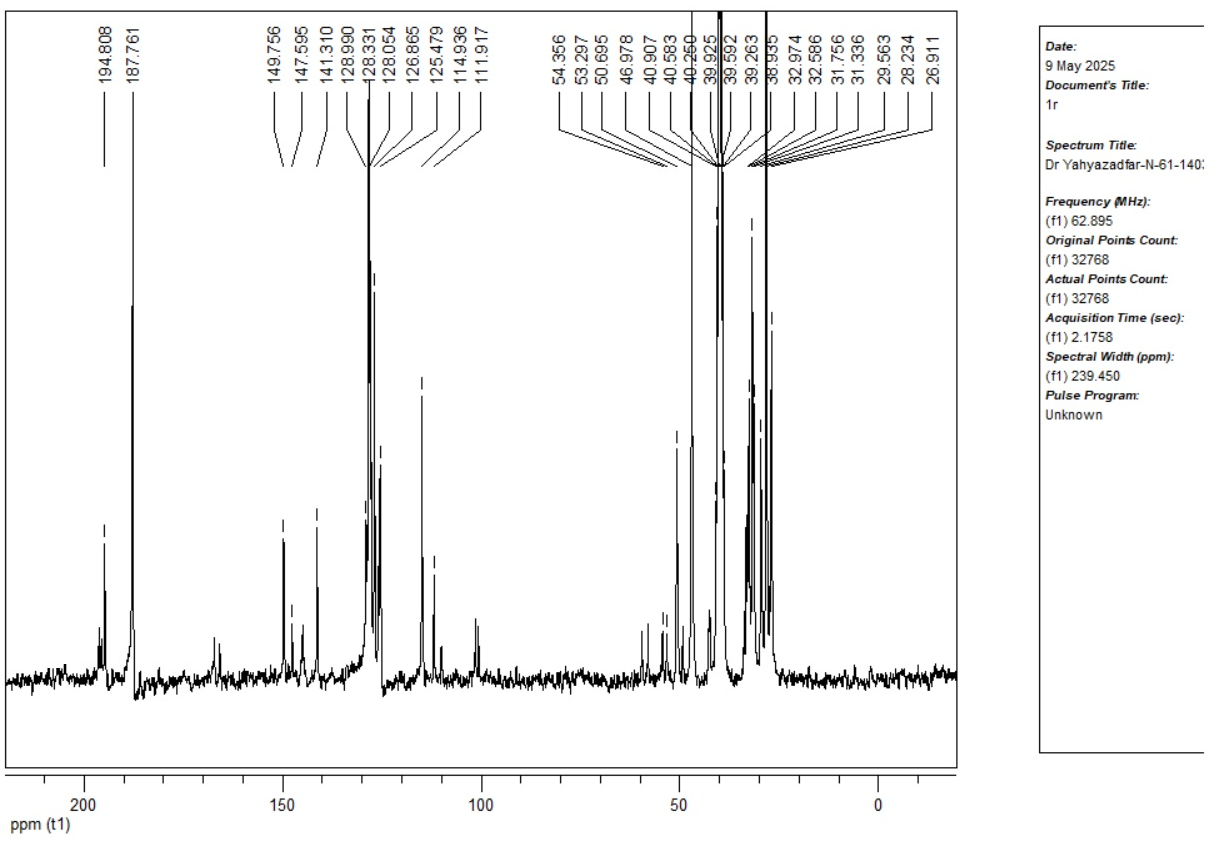


**Figure S-8.** ^13^C NMR spectrum of compound 4d.

**^1^H NMR and ^13^C NMR of compound (4e)**

*9-(4-chlorophenyl)-3,3,6,6-tetramethyl-3,4,6,7,9,10-hexahydroacridine-1,8(2H,5H)-dione (****4e****)*: Yield: 95%, m,p. = 227-230 ^°^C. ^1^H NMR (250 MHz, DMSO-*d_6_*, ppm) δ: 0.83 (s, 6H, 2CH_3_), 0.99 (s, 6H, 2CH_3_), 2.30 (brs, 8H, 4CH_2_), 4.24 (s, 1H, CH), 5.92 (brs, 1H, NH), 6.84-7.23 (m, 4H, H-Ar). ^13^C NMR (62.5 MHZ, DMSO-*d_6_*, ppm) δ: 26.9, 28.2, 29.5, 30.9, 31.7, 32.7, 33.0, 47.0, 50.7, 53.3, 54.3, 101.5, 114.6, 127.5, 128.2, 128.7, 130.0, 130.2, 130.8, 140.8, 144.2, 187.6, 195.7.

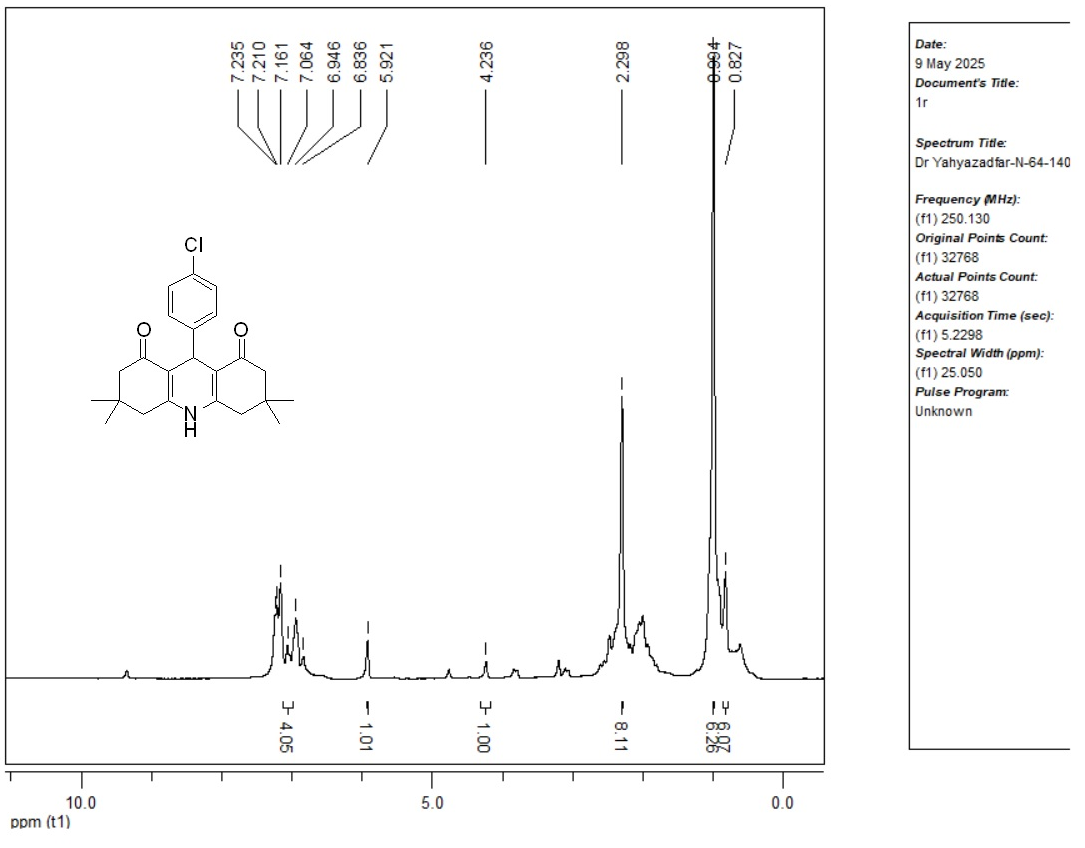


**Figure S-9.** ^1^H NMR spectrum of compound 4e.


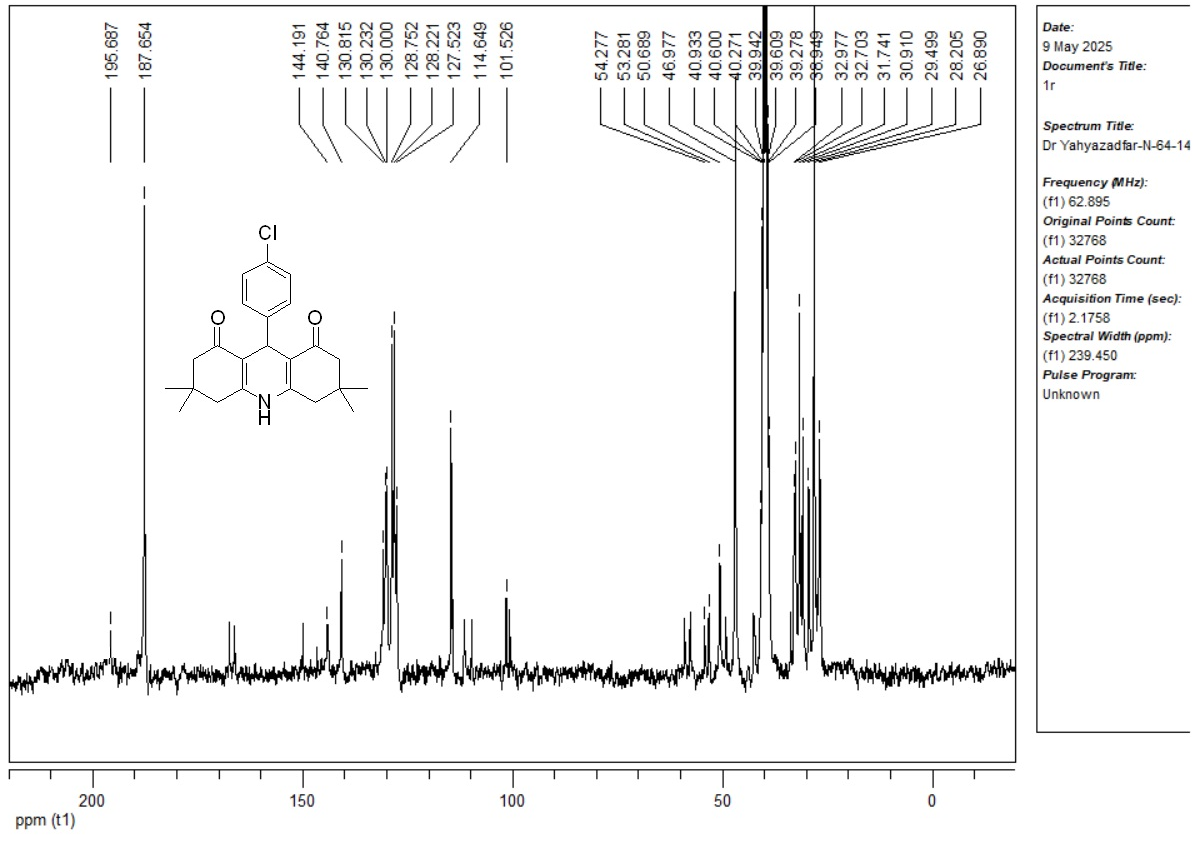


**Figure S-10.** ^13^C NMR spectrum of compound 4e.

**^1^H NMR and ^13^C NMR of compound (4f)**

3,3,6,6-tetramethyl-9-(4-nitrophenyl)-3,4,6,7,9,10-hexahydroacridine-1,8(2H,5H)-dione *(****4f****)*: Yield: 98%, M,p. = 280-282 ^°^C. ^1^H NMR (250 MHz, DMSO-*d_6_*, ppm) δ: 1.01 (s, 12H, 4CH_3_), 2.32 (brs, 8H, 4CH_2_), 4.35 (brs, 1H, CH), 6.08 (brs, 1H, NH), 7.31-7.43 (m, 2H, H-Ar), 8.01-8.08 (m, 2H, H-Ar). ^13^C NMR (62.5 MHZ, DMSO-*d_6_*, ppm) δ: 26.9, 28.2, 31.8, 32.3, 32.8, 33.0, 33.7, 46.9, 50.3, 50.6, 53.2, 101.6, 114.3, 122.9, 123.6, 128.1, 129.6, 130.3, 145.7, 151.1, 153.8, 187.6, 196.5.

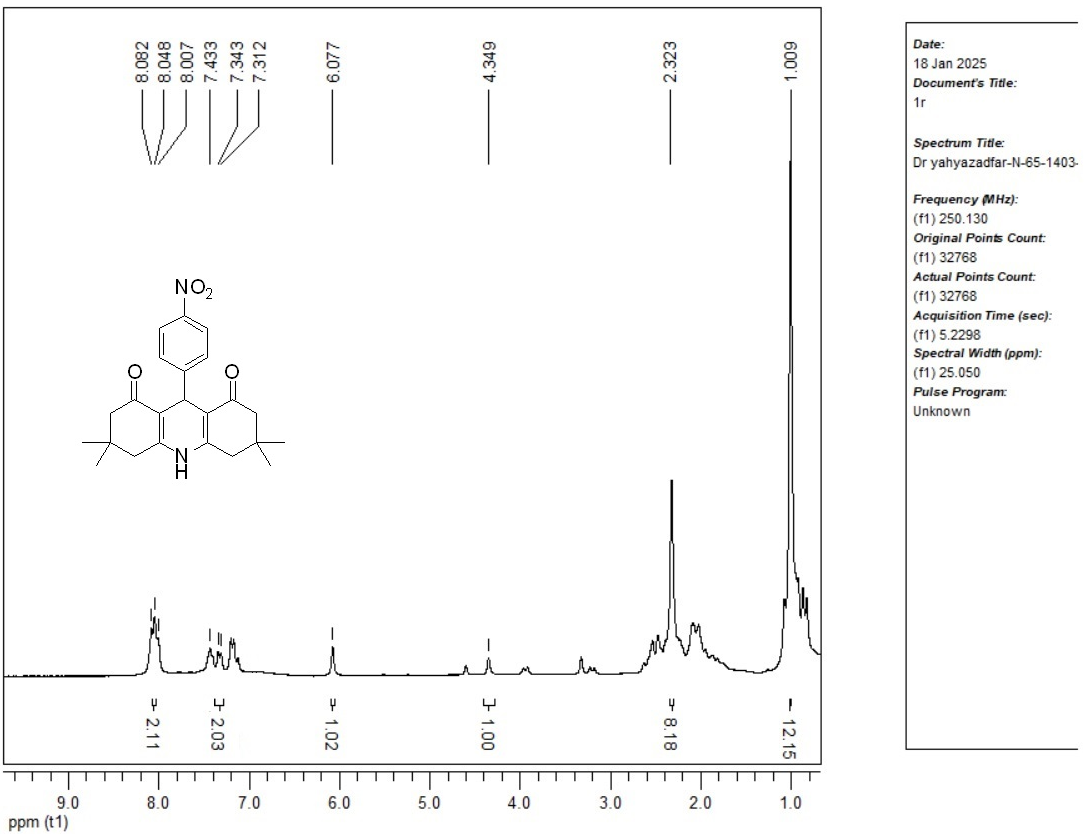


**Figure S-11.** ^1^H NMR spectrum of compound 4f.


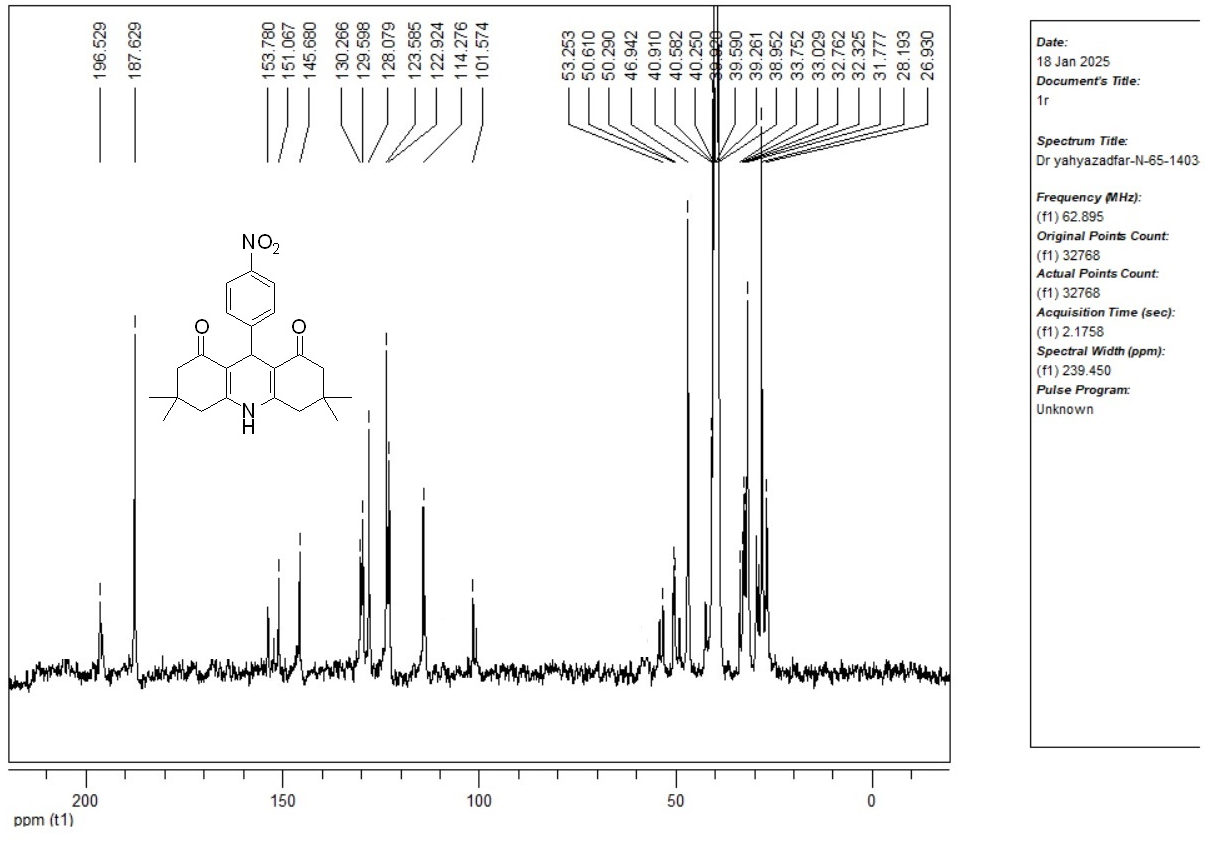


**Figure S-12.** ^13^C NMR spectrum of compound 4f.

**^1^H NMR and ^13^C NMR of compound (4g)**

*9-(4-methoxyphenyl)-3,3,6,6-tetramethyl-3,4,6,7,9,10-hexahydroacridine-1,8(2H,5H)-dione (****4g****)*: Yield: 98%, M,p. = 273-274 ^°^C. ^1^H NMR (250 MHz, DMSO-*d_6_*, ppm) δ: 0.84 (brs, 6H, 2CH_3_), 0.98 (s, 3H, CH_3_), 1.01 (s, 3H, CH_3_), 2.29 (brs, 8H, 4CH_2_), 4.72 (brs, 1H, CH), 6.69 (d, 1H, *J*= 8 Hz,H-Ar), 6.84 (d, 1H, *J*= 8.5 Hz, H-Ar), 7.03 (d, 2H, *J*= 7.75 Hz, H-Ar), 9.24 (brs, 1H, NH). ^13^C NMR (62.5 MHZ, DMSO-*d_6_*, ppm) δ: 26.9, 28.2, 29.5, 31.7, 32.3, 32.6, 32.9, 47.0, 50.7, 54.4, 55.3, 112.1, 113.3, 113.7, 115.2, 127.8, 128.9, 139.9, 149.5, 157.3, 157.5, 187.7, 194.8.

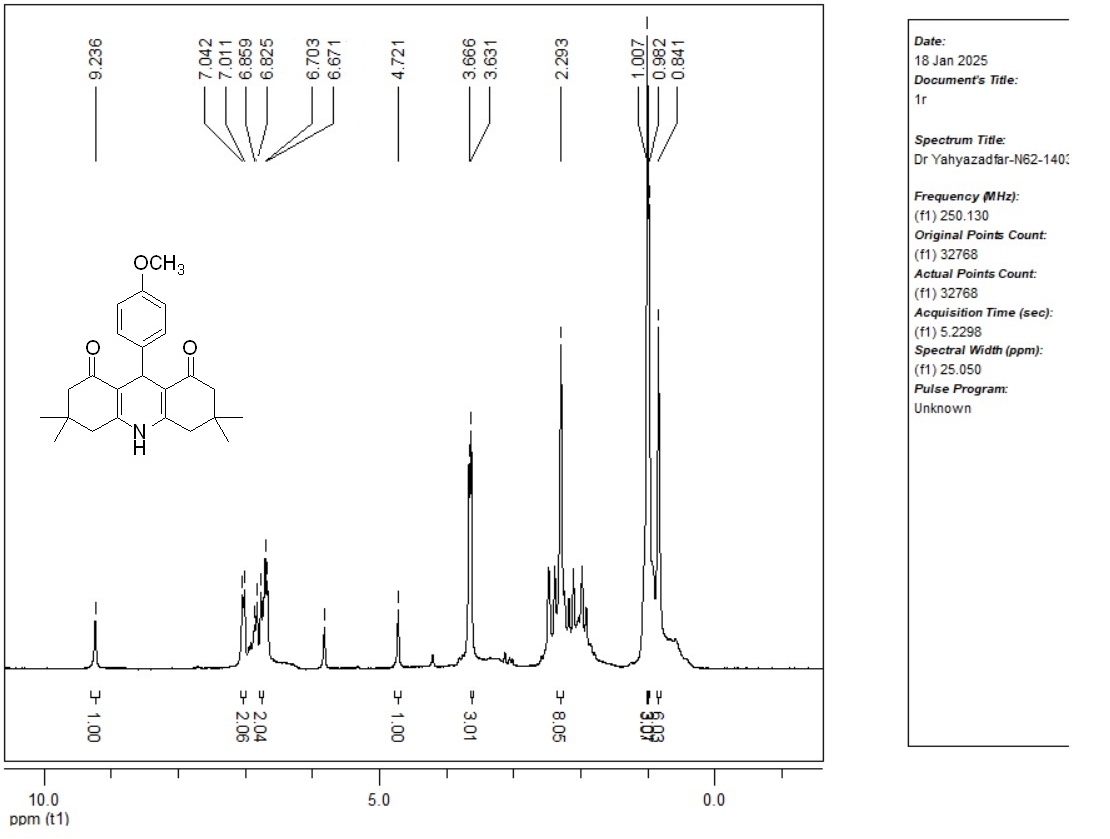


**Figure S-13.** ^1^H NMR spectrum of compound 4g.


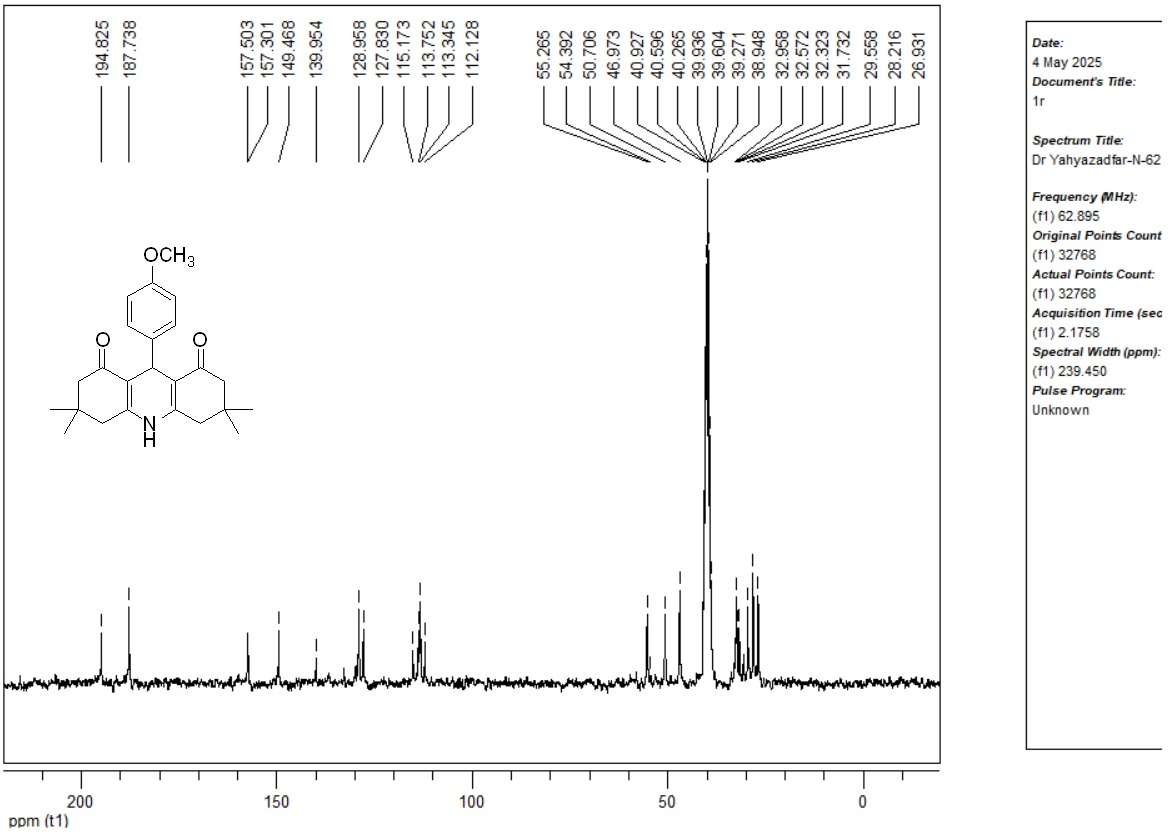


**Figure S-14.** ^13^C NMR spectrum of compound 4g.

**^1^H NMR and ^13^C NMR of compound (4h)**

*9-(2,4-dichlorophenyl)-3,3,6,6-tetramethyl-3,4,6,7,9,10-hexahydroacridine-1,8(2H,5H)-dione (****4h****)*: Yield: 97%, m,p. = 317-320 ^°^C. ^1^H NMR (250 MHz, DMSO-*d_6_*, ppm) δ: 0.90 (s, 3H, CH_3_), 0.95 (s, 3H, CH_3_), 1.00 (s, 3H, CH_3_), 1.05 (s, 3H, CH_3_), 2.04 (brs, 2H, CH_2_), 2.11 (brs, 2H, CH_2_), 2.22-2.48 (m, 4H, 2CH_2_), 4.49 (s, 1H, CH), 7.01 (d, *J*= 2 Hz, 1H, H-Ar), 7.07 (d, *J*= 2 Hz, 1H, H-Ar), 7.14 (t, *J*= 2 Hz, 1H, H-Ar), 7.45 (s, 1H, NH). ^13^C NMR (62.5 MHZ, DMSO-*d_6_*, ppm) δ: 26.6, 26.7, 28.2, 29.0, 29.5, 32.6, 33.1, 47.1, 49.0, 50.7, 55.0, 101.6, 109.1, 114.0, 126.4, 128.3, 131.0, 132.8, 133.3, 140.5, 168.2, 186.2, 196.1.

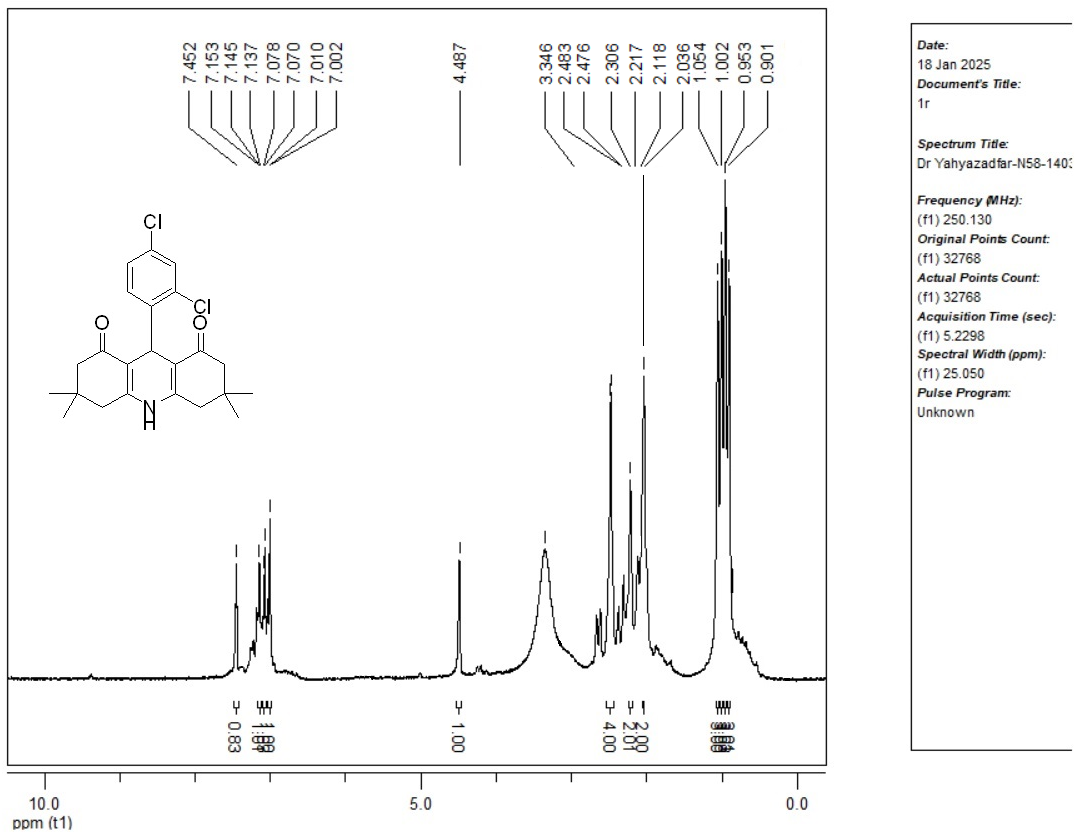


**Figure S-15.** ^1^H NMR spectrum of compound 4h.


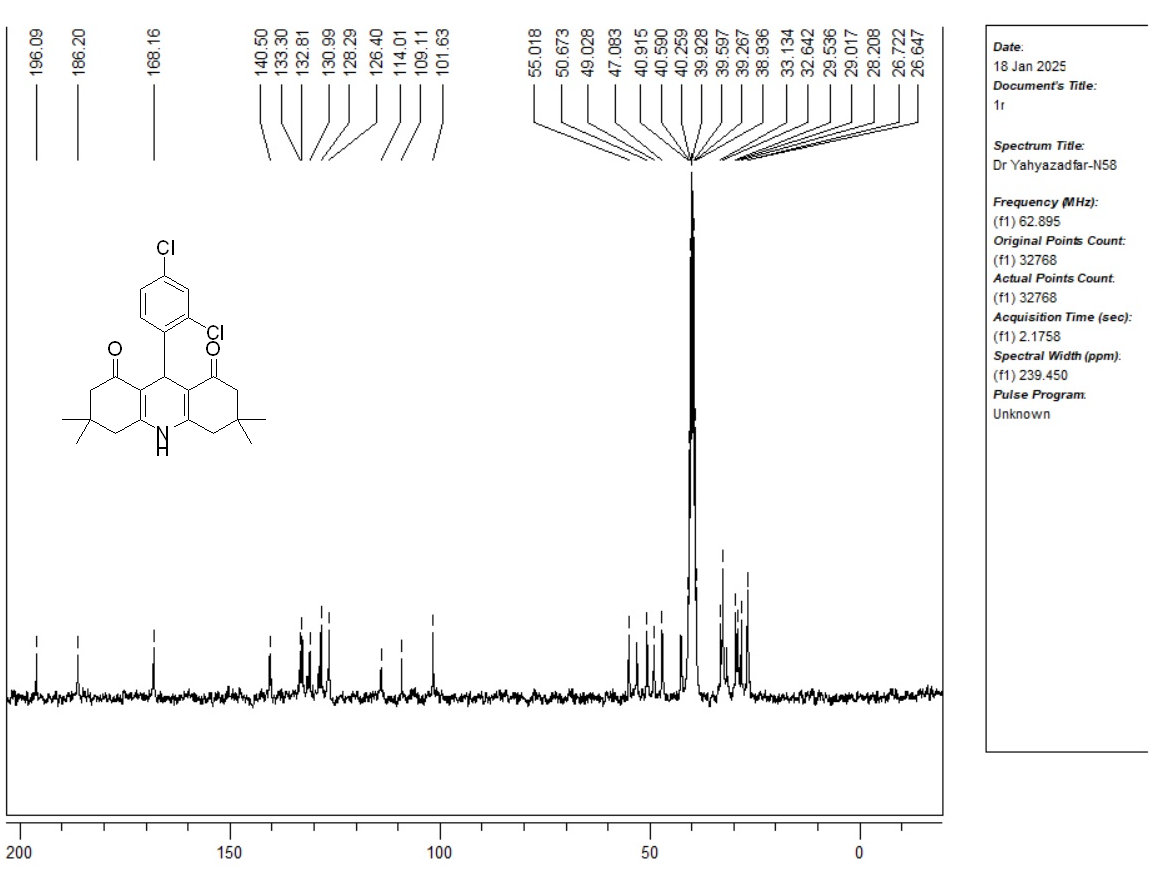


**Figure S-16.** ^13^C NMR spectrum of compound 4h.

**^1^H NMR and ^13^C NMR of compound (4i)**

*9-(2-methoxyphenyl)-3,3,6,6-tetramethyl-3,4,6,7,9,10-hexahydroacridine-1,8(2H,5H)-dione (****4i****)*: Yield: 98%, m,p. = 297-298 ^°^C. ^1^H NMR (250 MHz, DMSO-*d_6_*, ppm) δ: 0.82 (s, 3H, CH_3_), 0.92 (s, 3H, CH_3_), 0.98 (s, 3H, CH_3_), 1.07 (s, 3H, CH_3_), 1.98-2.35 (m, 8H, 4CH_2_), 3.70 (s, 1H, OCH_3_), 4.49 (s, 1H, CH), 6.66-6.74 (m, 3H, 1NH, 2H-Ar), 6.85 (t, *J*= 7.25 Hz, 1H, H-Ar), 7.03 (t, *J*= 7.75 Hz, 1H, H-Ar). ^13^C NMR (62.5 MHZ, DMSO-*d_6_*, ppm) δ: 26.3, 26.9, 28.1, 29.4, 31.7, 32.6, 33.1, 47.0, 49.2, 50.9, 53.3, 55.6, 101.6, 110.3, 115.4, 119.6, 126.5, 128.4, 129.6, 128.4, 129.6, 128.4, 129.6, 131.6, 156.5, 167.4, 186.4, 196.0.

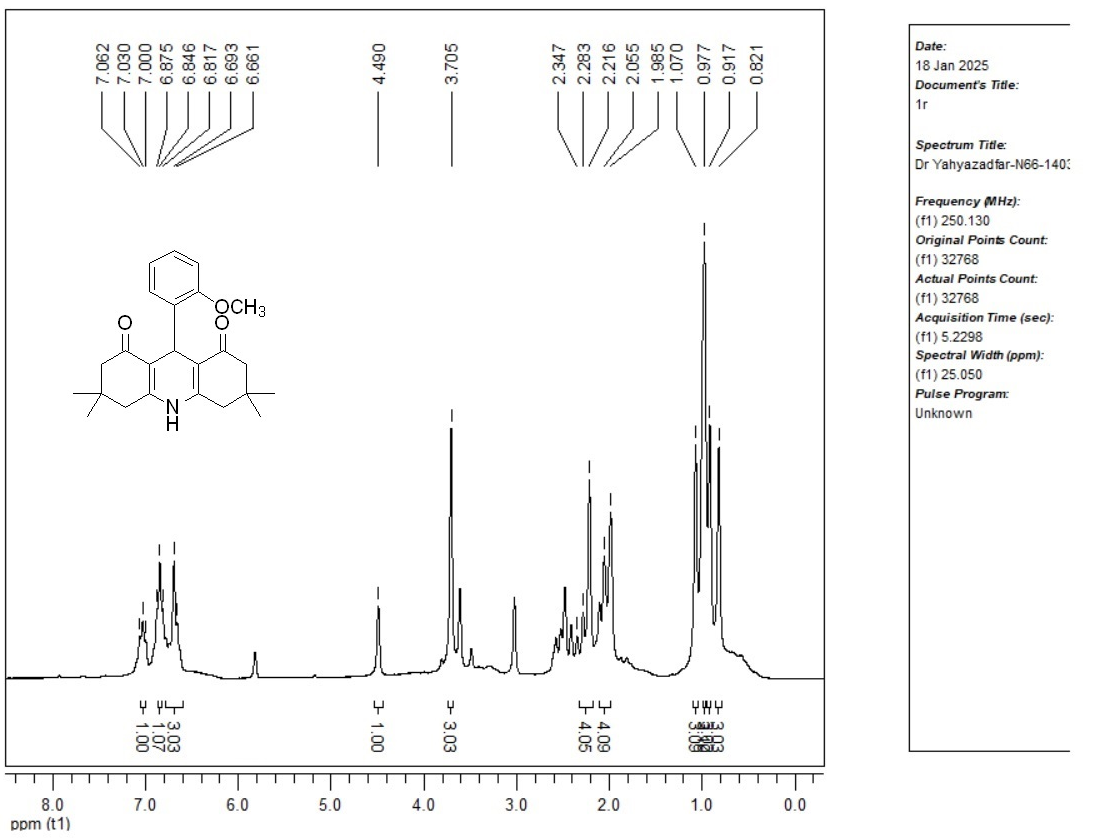


**Figure S-17.** ^1^H NMR spectrum of compound 4i.


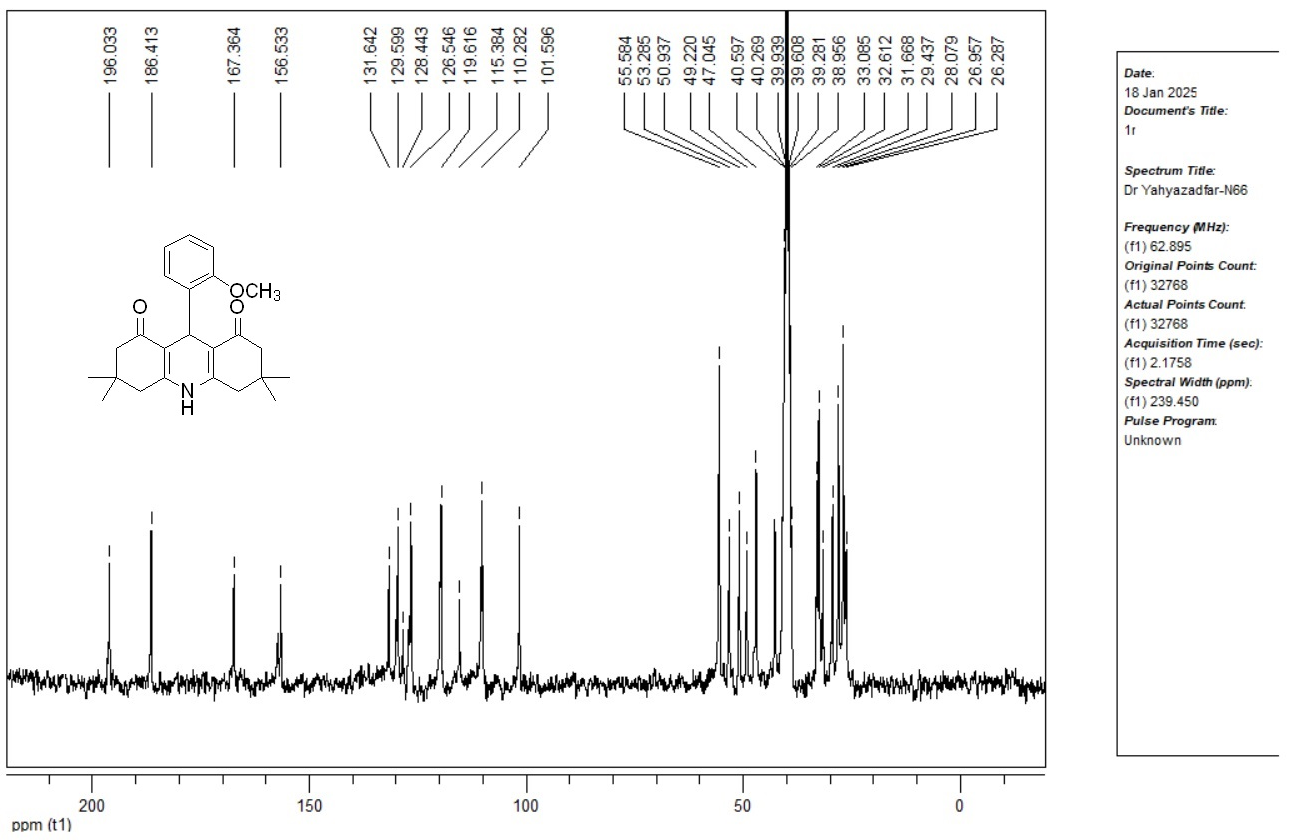


**Figure S-18.** ^13^C NMR spectrum of compound 4i.

**^1^H NMR and ^13^C NMR of compound (4j)**

*9-(3-chlorophenyl)-3,3,6,6-tetramethyl-3,4,6,7,9,10-hexahydroacridine-1,8(2H,5H)-dione (****4j****):* Yield: 95%. M,p. = 195-197 ^°^C. ^1^H NMR (250 MHz, DMSO-*d_6_*, ppm) δ: 0.83 (s, 3H, CH_3_), 0.95 (s, 3H, CH_3_), 1.03 (s, 3H, CH_3_), 1.10 (s, 3H, CH_3_), 2.34 (brs, 8H, 4CH_2_), 4.36 (s, 1H, CH), 6.08 (brs, 1H, NH), 7.41-7.78 (m, 3H, H-Ar), 7.95 (s, 1H, H-Ar). ^13^C NMR (62.5 MHZ, DMSO-*d_6_*, ppm) δ: 26.8, 28.2, 29.0, 31.3, 31.8, 32.7, 33.0, 47.0, 50.6, 53.3, 57.4, 113.8, 120.6, 121.4, 123.2, 129.1, 129.9, 133.8, 144.6, 147.5, 148.2, 187.8, 196.6.

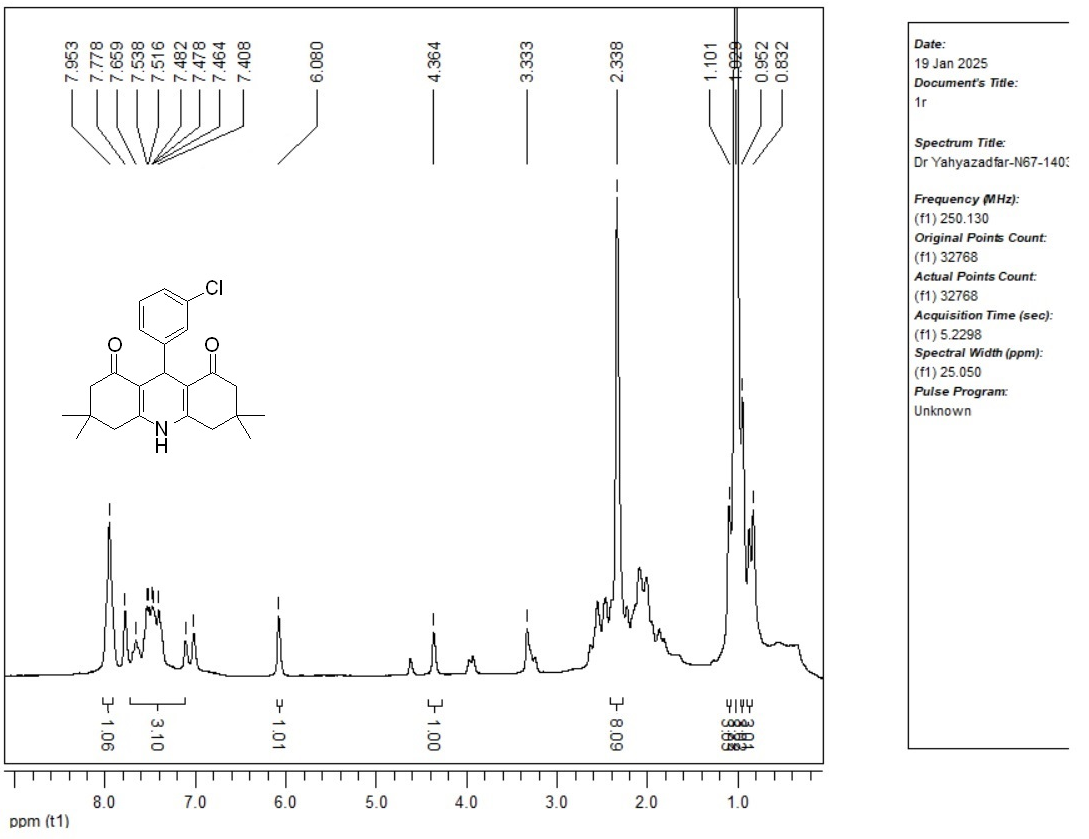


**Figure S-19.** ^[1](https://www.google.com/url?sa=t&rct=j&q=&esrc=s&source=web&cd=&cad=rja&uact=8&ved=2ahUKEwjmjuPvw579AhW_8LsIHTvtAd8QFnoECBAQAQ&url=https%3A%2F%2Fwww.researchgate.net%2Ffigure%2FThe-1-H-NMR-spectrum-of-compound-3a-in-CDCl-3-solvent_fig1_320865085&usg=AOvVaw2S68h8geITZVHy7tcJjbIy)^[H NMR spectrum of compound 4j.](https://www.google.com/url?sa=t&rct=j&q=&esrc=s&source=web&cd=&cad=rja&uact=8&ved=2ahUKEwjmjuPvw579AhW_8LsIHTvtAd8QFnoECBAQAQ&url=https%3A%2F%2Fwww.researchgate.net%2Ffigure%2FThe-1-H-NMR-spectrum-of-compound-3a-in-CDCl-3-solvent_fig1_320865085&usg=AOvVaw2S68h8geITZVHy7tcJjbIy)


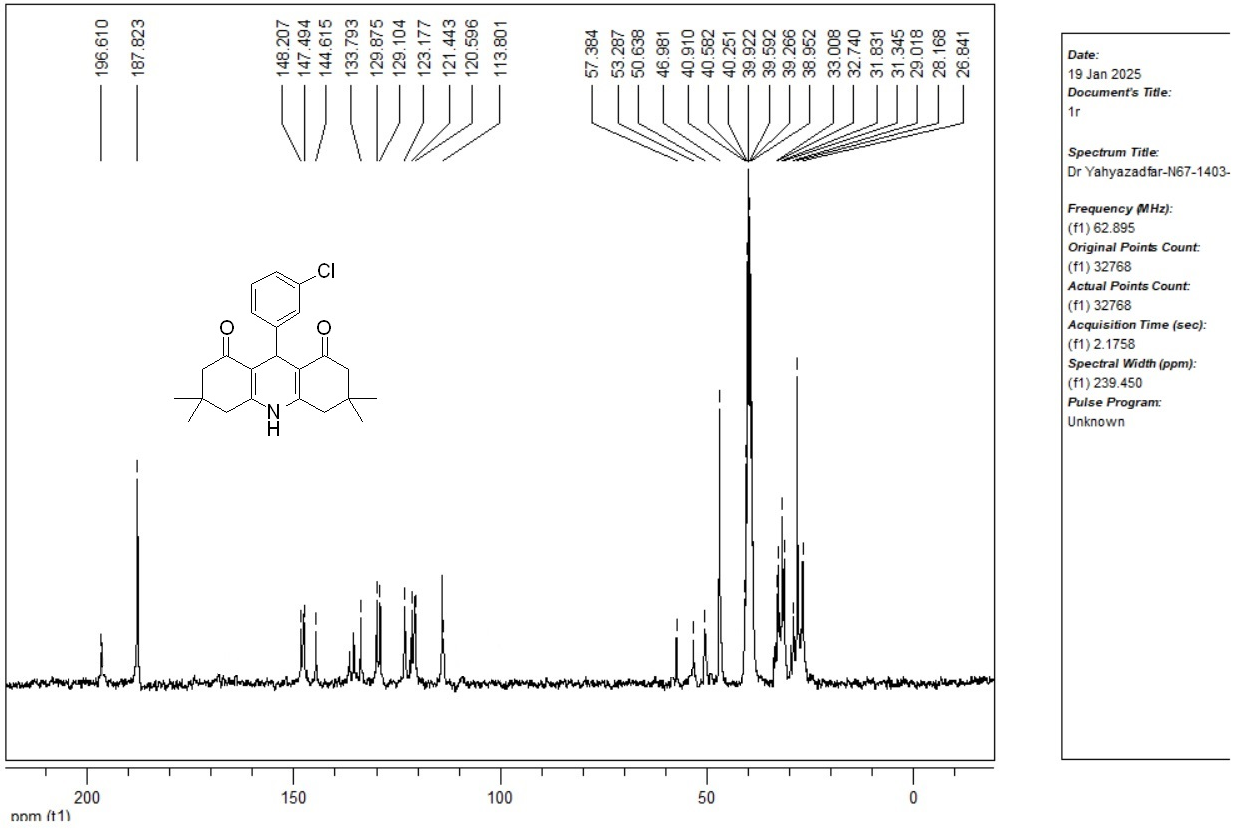


**Figure S-20.** ^13^C NMR spectrum of compound 4j.

**^1^H NMR and ^13^C NMR of compound (4k)**

*9-(2-hydroxynaphthalen-1-yl)-3,3,6,6-tetramethyl-3,4,6,7,9,10-hexahydroacridine-1,8(2H,5H)-dione (****4k****)*: Yield: 95%, M,p. = 175-177 ^°^C. ^1^H NMR (400 MHz, DMSO-*d_6_*, ppm) δ: 0.48 (s, 3H, CH_3_), 0.72 (s, 3H, CH_3_), 0.84 (s, 3H, CH_3_), 0.93 (s, 3H, CH_3_), 1.39 (brs, 1H, NH), 1.66 (dd, 2H, *J_1_*= 6.4 Hz, *J_2_*= 53.2 Hz, CH_2_), 2.17-2.47 (m, 6H, 3CH_2_), 5.04 (s, 1H, CH), 7.03-7.05 (m, 2H, H-Ar), 7.15 (t, 1H, J= 7.4 Hz, H-Ar), 7.22-7.26 (m, 1H, H-Ar), 7.49 (d, *J*= 8.8 Hz, 2H, H-Ar), 7.55 (d, *J*= 7.6 Hz, 1H, H-Ar), 10.46 (brs, 1H, OH). ^13^C NMR (100 MHZ, DMSO-*d_6_*, ppm) δ: 26.8, 28.2, 29.0, 31.3, 31.8, 32.7, 33.0, 47.0, 50.6, 53.3, 57.4, 113.8, 120.6, 121.4, 123.2, 129.1, 129.9, 133.8, 144.6, 147.5, 148.2, 187.8, 196.6.

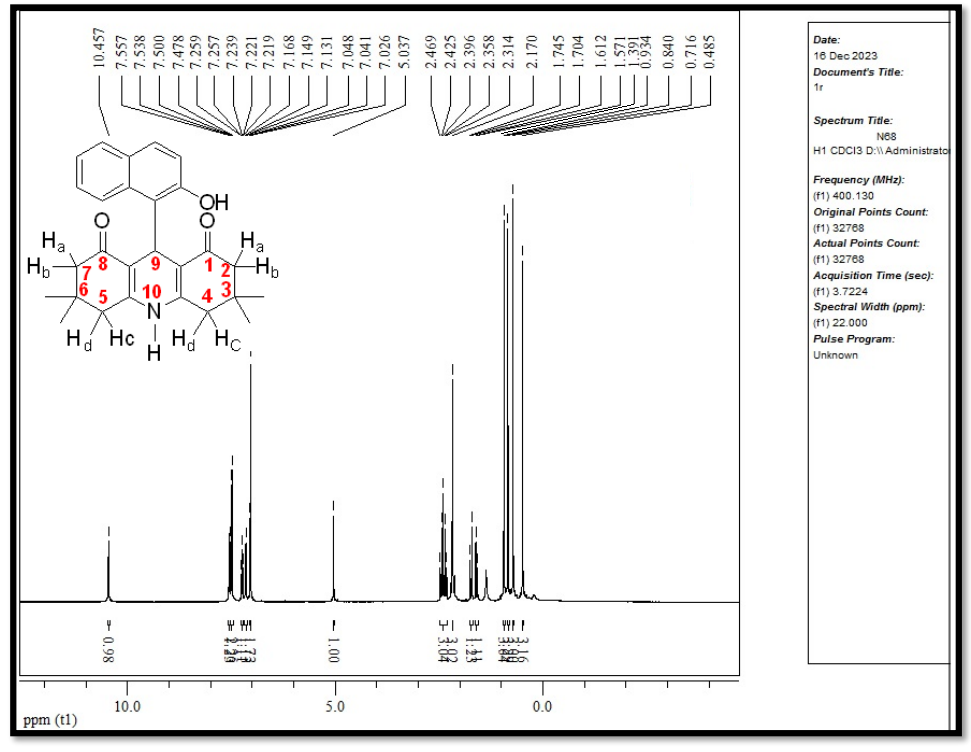


**Figure S-21.** ^1^H NMR spectrum of compound 4k.


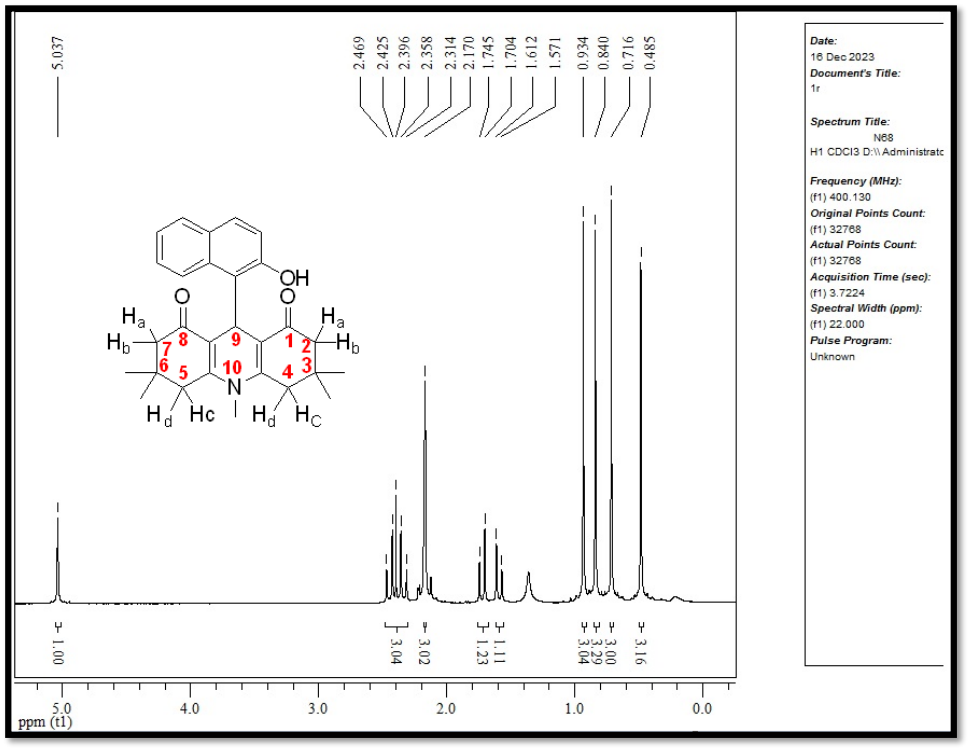


**Figure S-21a.** ^1^H NMR expand spectrum of compound 4k.


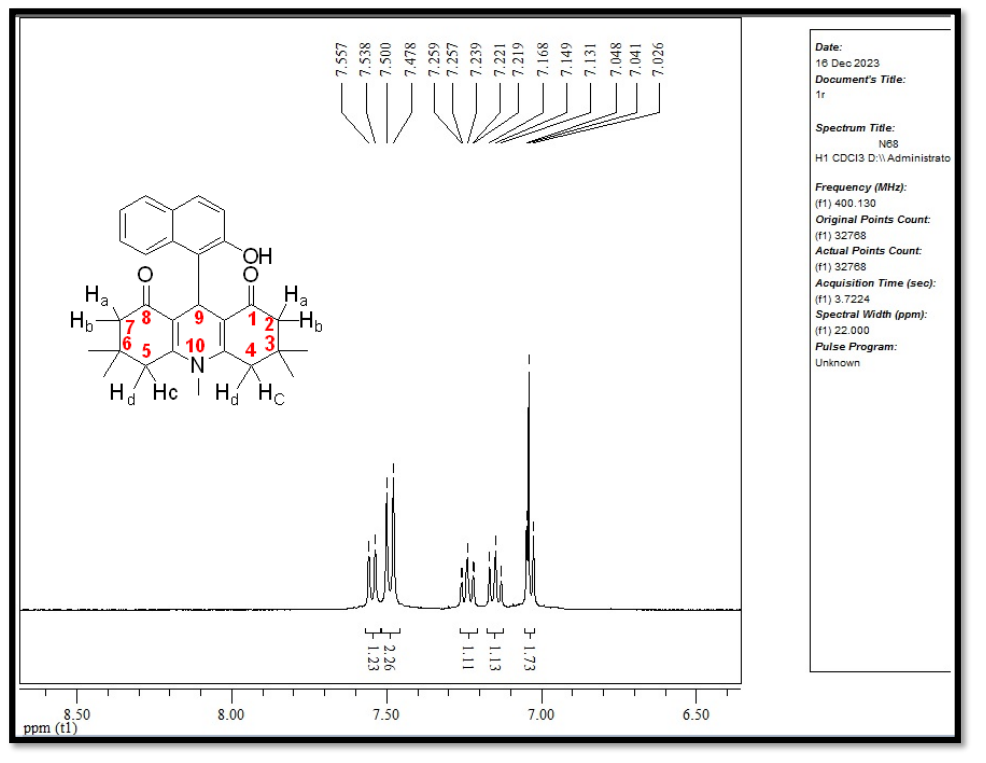


**Figure S-21b.** ^1^H NMR expand spectrum of compound 4k.


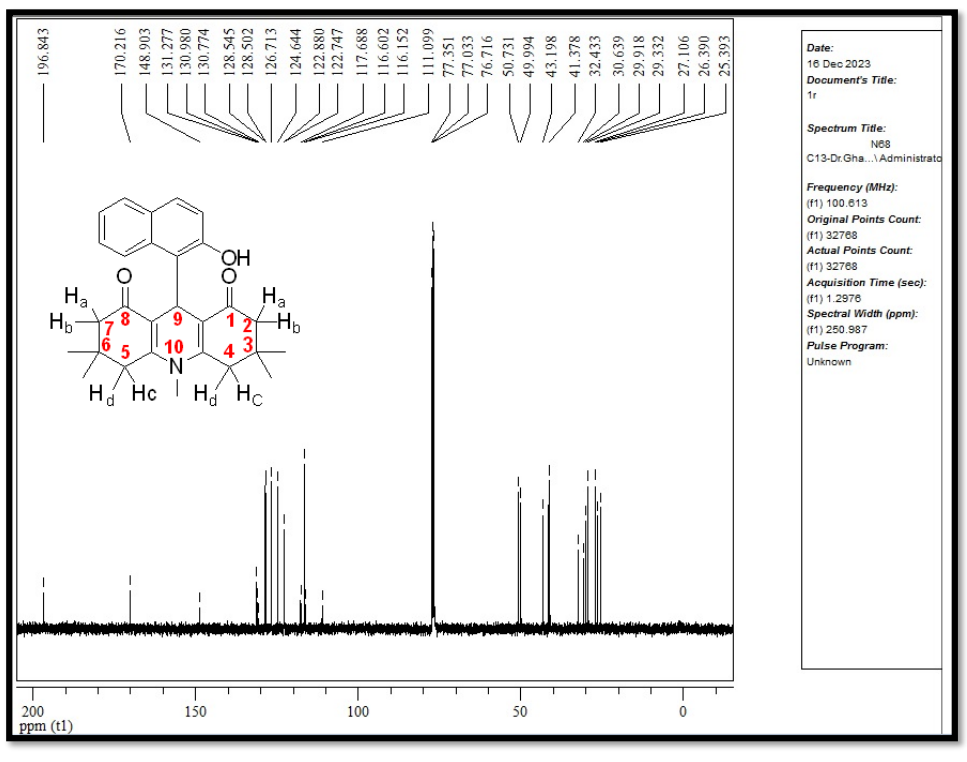


**Figure S-22.** ^13^C NMR expand spectrum of compound 4k.

**^1^H NMR and ^13^CNMR of compound (4l)**

*9-(2,4-dimethoxyphenyl)-3,3,6,6-tetramethyl-3,4,6,7,9,10-hexahydroacridine-1,8(2H,5H)-dione (****4l****)*: Yield: 99%, m,p. = 198-201 ^°^C. ^1^H NMR (250 MHz, DMSO-*d_6_*, ppm) δ: 0.81 (s, 3H, CH_3_), 0.90 (s, 3H, CH_3_), 0.96 (s, 3H, CH_3_), 1.06 (s, 3H, CH_3_), 1.97-2.48 (m, 8H, 4CH_2_), 3.66 (s, 3H, OCH_3_), 3.68 (s, 3H, OCH_3_), 4.39 (s, 1H, CH), 6.26-6.74 (m, 3H, H-Ar), 9.15 (brs, 1H, NH). ^13^C NMR (62.5 MHZ, DMSO-*d_6_*, ppm) δ: 27.0, 28.0, 29.3, 31.6, 32.4, 32.6, 33.0, 47.0, 49.2, 50.9, 53.2, 55.4, 55.7, 97.9, 101.6, 104.0, 110.2, 115.5, 123.8, 129.8, 157.2, 158.6, 167.2, 186.4, 196.0.

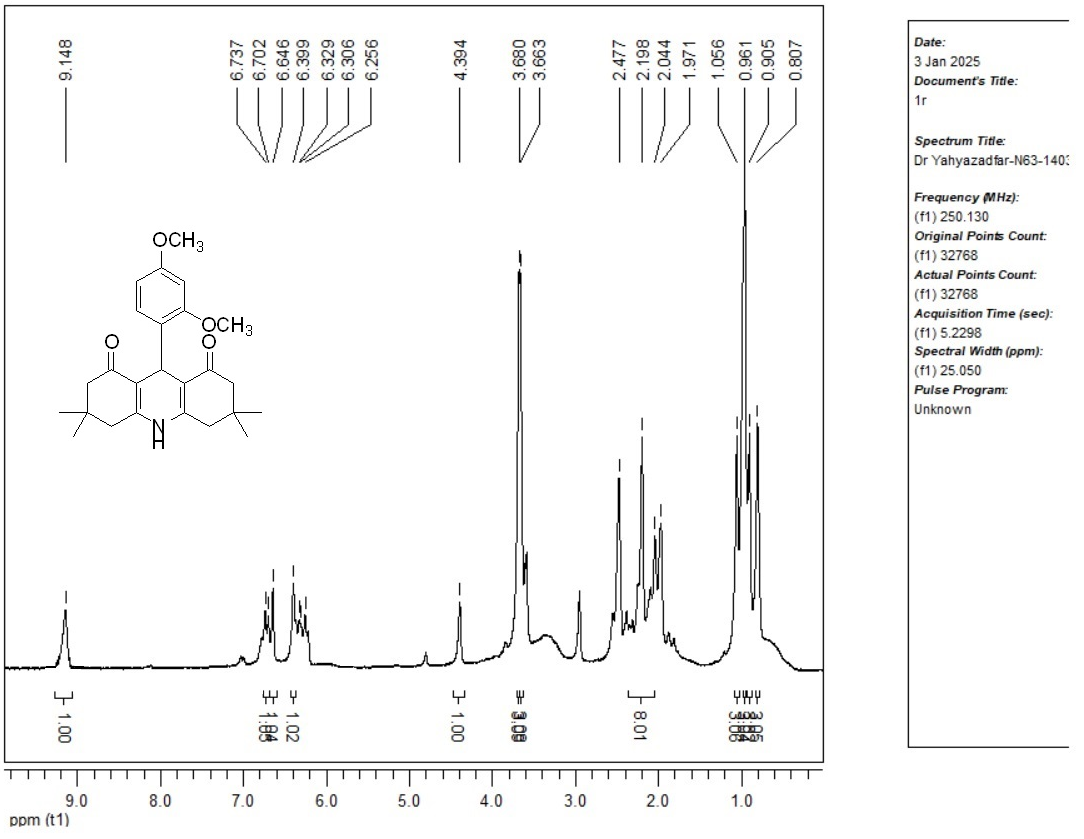


**Figure S-23.** ^1^H NMR spectrum of compound 4l.


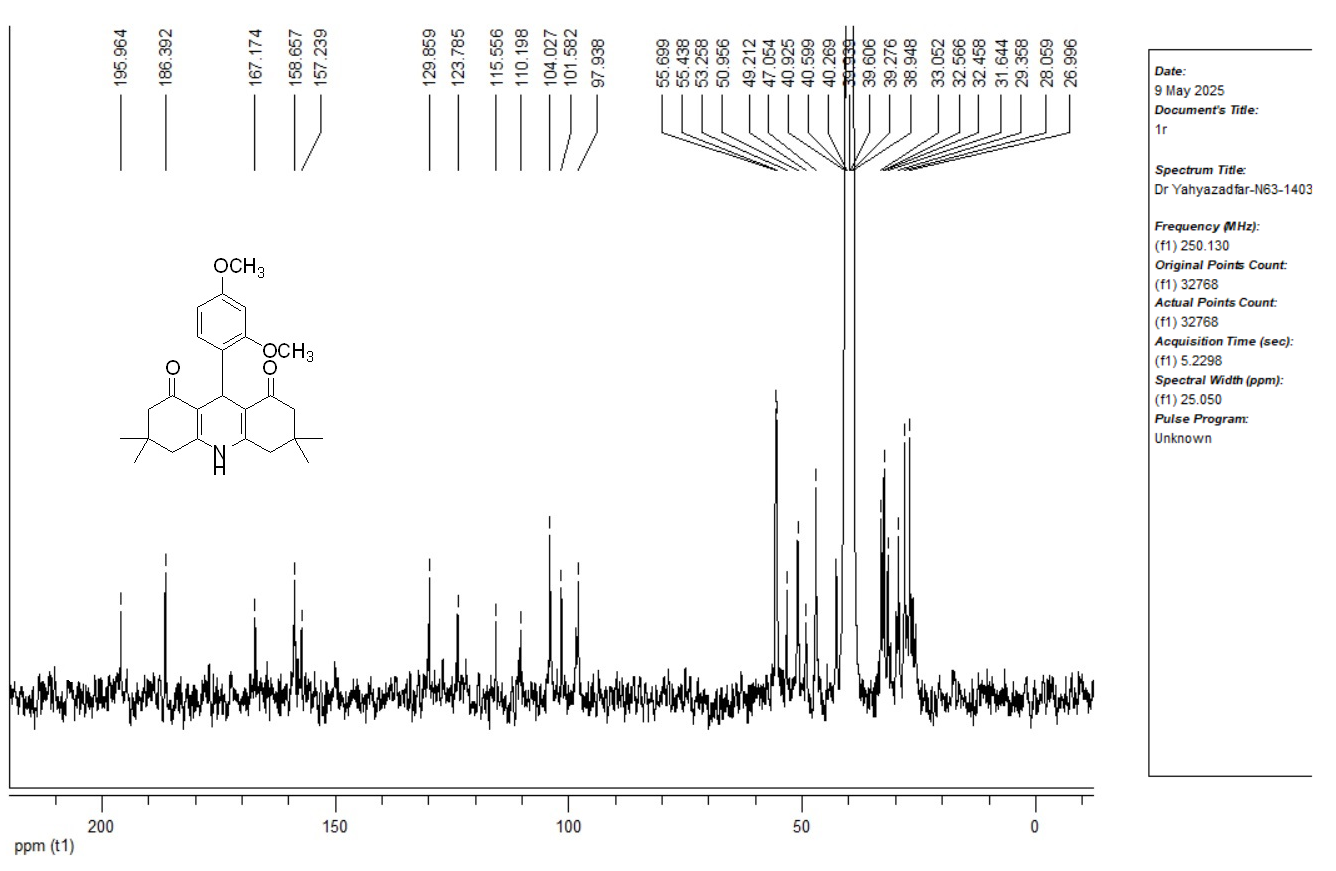


**Figure S-24.** ^13^C NMR spectrum of compound 4l.


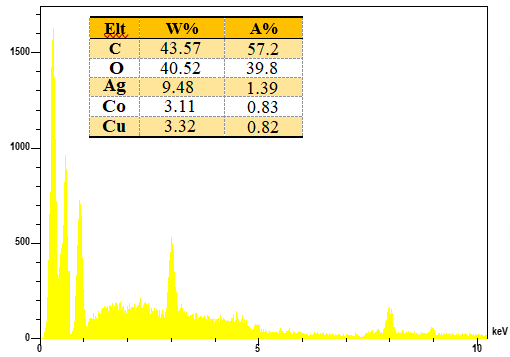


**Figure S-25.** EDX elemental analysis of BF Co-Ag MOF@CuO nano-catalytic material after recycled procedure.


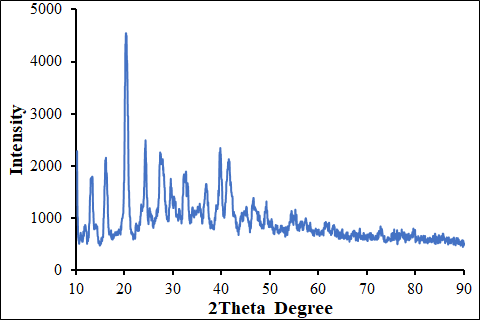


**Figure S-26.** XRD analysis of Co-Ag MOF@CuO nano-catalytic material after recycled procedure.
